# Convenient and efficient synthesis of functionalized unsymmetrical *Z*-alkenyl disulfanes†

**DOI:** 10.1039/c8ra00659h

**Published:** 2018-03-07

**Authors:** M. Musiejuk, J. Doroszuk, D. Witt

**Affiliations:** Department of Organic Chemistry, Faculty of Chemistry, Gdansk University of Technology Narutowicza 11/12 80-233 Gdansk Poland chemwitt@pg.gda.pl +48 58 3472694

## Abstract

We developed a simple and efficient method for the synthesis of functionalized unsymmetrical *Z*-alkenyl disulfanes under mild conditions in moderate to good yields. The designed method is based on the reaction of *Z*-alkenyl thiotosylates with thiols in the presence of base. The developed method allows the preparation of unsymmetrical *Z*-alkenyl disulfanes bearing additional hydroxy, carboxy, or amino functionalities.

## Abstract 2

We developed a simple and efficient method for the synthesis of functionalized unsymmetrical *Z*-alkenyl disulfanes under mild conditions in moderate to good yields.

## Graphical abstract

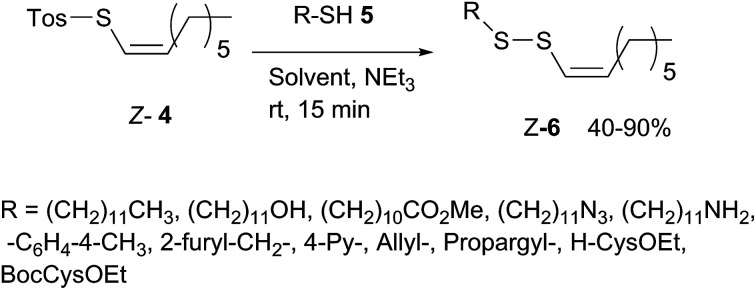

## Introduction

Compounds with R-S-S-R structures, where the R groups are alkyl, vinyl or aryl, are known as symmetrical disulfides if the R groups are the same. A large number of unsymmetrical disulfides, in which the R groups are different, are also well known. In the literature, these compounds are often called organic disulfides; however, the IUPAC recommended nomenclature is disulfanes.^1^ The name disulfide should only be applied to ionic compounds, such as sodium disulfide (Na_2_S_2_). Moreover, the term disulfane is more widely applicable than disulfide because it facilitates naming even when the R groups are acyl and/or phosphoryl groups.

The formation of unsymmetrical disulfanes is an important transformation in organic synthesis and medicinal chemistry.^2^ Recent developments in disulfide bond formation reactions have been reviewed.^3^ Although many different methods exist for the preparation of unsymmetrical disulfanes, the most common approach involves substitution of a sulfenyl derivative with a thiol or thiol derivative. To date, the most commonly utilized sulfenyl derivatives are sulfenyl chlorides,^4^*S*-alkyl thiosulfates and *S*-aryl thiosulfates (Bunte salts),^5^*S*-alkylsulfanylisothioureas,^6^ benzothiazol-2-yl disulfanes,^7^ benzotriazolyl sulfanes,^8^ dithioperoxyesters,^9^ (alkylsulfanyl)dialkylsulfonium salts,^10^ 2-pyridyl disulfanes and derivatives,^11^*N*-alkyltetrazolyl disulfanes,^12^ sulfenamides,^13^ sulfenyldimesylamines,^14^ sulfenyl thiocyanates,^15^ 4-nitroarenesulfenanilides,^16^ thiolsulfinates and thiosulfonates,^17^ sulfanylsulfinamidines,^18^ thionitrites,^19^ sulfenyl thiocarbonates,^20^ thioimides,^21^ and thiophosphonium salts.^22^ Other practical procedures involve the reaction of a thiol with a sulfinylbenzimidazole,^23^ a rhodium-catalyzed disulfide exchange,^24^ an electrochemical method,^25^ the ring opening of an aziridine using tetrathiomolybdate in the presence of a symmetrical disulfane,^26^ or the use of diethyl azodicarboxylate (DEAD)^27^ or a solid support^28^ in a sequential coupling of two different thiol groups. Recently, the oxidation of a mixture of two different thiols by 2,3-dichloro-5,6-dicyanobenzoquinone (DDQ) to produce an unsymmetrical disulfane has also been reported.^29^

Earlier studies demonstrated the preparation of functionalized unsymmetrical molecules, such as dialkyl disulfanes,^30^ alkyl-aryl disulfanes,^31^ ‘bioresistant’ disulfanes,^32^ the unsymmetrical disulfanes of l-cysteine and l-cystine,^33^ and diaryl disulfanes^34^ based on the readily available 5,5-dimethyl-2-thioxo-1,3,2-dioxaphosphorinane-2-disulfanyl derivatives. These disulfanyl derivatives of phosphorodithioic acid were convenient for the preparation of α-sulfenylated carbonyl compounds,^35^ functionalized phosphorothioates,^36^ and unsymmetrical alkynyl sulfides^37^ as well as symmetrical^38^ and unsymmetrical^39^ trisulfanes.

Ajoene was first isolated by Block^40^ in 1984 as an *E*/*Z*-mixture of a rearrangement product of allicin produced from freshly crushed garlic. It was established to be an allyl sulfoxide containing an unusual vinyl disulfane functionality, which is rarely seen in the structures of natural products. *Z*-Ajoene is more active than its *E*-isomer as an anti-thrombotic agent,^41^ and some studies on anticancer treatments have focused primarily on the *Z*-isomer.^42^

Although many different synthetic methods exist for the preparation of unsymmetrical disulfanes, the preparation of unsymmetrical alkenyl disulfanes can be achieved by only two methods. The first method is based on the reaction of sulfenyl bromide with trityl-alkenyl sulfide.^43^ The second method involves the low temperature hydroxide-promoted cleavage of an alkenyl thioester followed by sulfenylation with an appropriate *S*-alkylated *p*-toluenethiosulfonate to afford vinyl disulfide in high yield after chromatography.^44^ Unfortunately, the methods provide exclusively *E* or a mixture of *Z*/*E* alkenyl disulfanes, respectively. In this context, we set out to investigate the feasibility of a more convenient and experimentally practical diastereoselective method to exclusively access *Z*-alkenyl disulfanes.

## Results and discussion

**Scheme 1:**
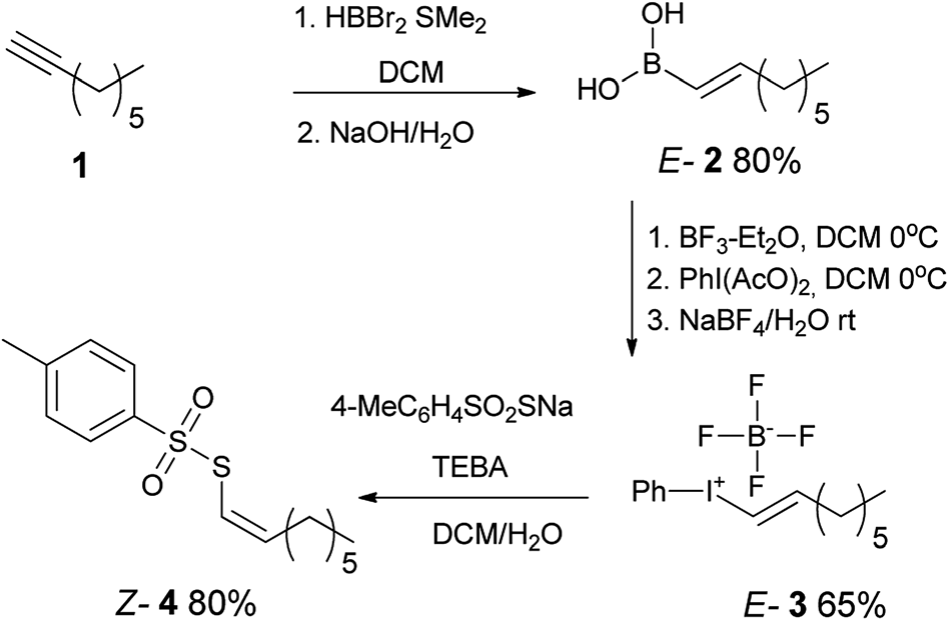
Preparation of *Z*-1-octenyl *p*-toluenethiosulfonate 4.

Our synthetic strategy included the preparation of *E*-alkenylboronic acid^45^2 from terminal alkyne 1 followed by its conversion to appropriate *E*-alkenyliodonium salt^46^3 by known methods. Further reaction with sodium *p*-toluenethiosulfonate provided *Z*-1-octenyl *p*-toluenethiosulfonate 4 with inversion of configuration (Scheme 1).

The reaction of *Z*-1-octenyl *p*-toluenethiosulfonate 4 with a variety of thiols in the presence of NEt_3_ provided *Z*-alkenyl disulfanes 6 in good or very good yield (Table 1). All compounds have been fully characterized by ^1^H and ^13^C NMR spectroscopy (see the ESI†). *E*/*Z*-Stereochemistry was assigned based on the vinyl coupling constants in the ^1^H NMR spectra; 15 Hz was indicative of the *E*-isomer and 10 Hz for the *Z*-isomer.

**Table 1:** Preparation of functionalized unsymmetrical *Z*-alkenyl disulfanes 6^a^

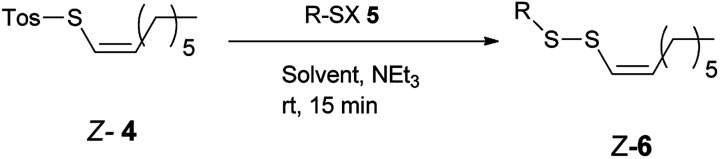					
Entry		R	X	Solvent	Yield^b^ (%)
1	5a	–C_12_H_25_	H	CH_2_Cl_2_	6a (90)
2	5b	–(CH_2_)_11_OH	H	CH_2_Cl_2_	6b (82)
3	5c	(CH_2_)_10_CO_2_Me	H	CH_2_Cl_2_	6c (88)
4	5d	–(CH_2_)_11_N_3_	H	CH_2_Cl_2_	6d (70)
5	5e	–(CH_2_)_11_NH_2_	H	CH_2_Cl_2_	6e (77)
6	5f	4-MeC_6_H_4_-	H	CH_2_Cl_2_	6f (40)
7	5f	4-MeC_6_H_4_-	H	CH_2_Cl_2_	6f (62)^c^
8	5g	2-Furyl–CH_2_–	H	CH_2_Cl_2_	6g (71)
9	5h	4-Py–	H	CH_2_Cl_2_	6h (52)
10	5i	CH_2_=CHCH_2_-	H	CH_2_Cl_2_	6i (51)
11	5i	CH_2_=CHCH_2_-	Ac	MeOH	6i (80)
12	5j	HC≡CCH_2_-	Ac	MeOH	6j (78)
13	5k	H-CysOEt	H	CH_2_Cl_2_	6k (60)
14	5l	BocCysOEt	H	CH_2_Cl_2_	6l (59)
15	5m	4-MeOC_6_H_4_-	H	CH_2_Cl_2_	6m (43)
16	5m	4-MeOC_6_H_4_-	H	CH_2_Cl_2_	6m (62)^c^

a Performed with 4 (0.67 mmol), 5 (0.61 mmol), NEt_3_ (0.61 mmol) in solvent (5 mL), 15 min.

b isolated yield.

c Performed with 4 (1.22 mmol), 5 (0.61 mmol), NEt_3_ (0.61 mmol) in solvent (5 mL), 15 min.

The reaction proceeded *via* the nucleophilic substitution of the thiolate anion (generated from 5) at the sulfur atom of thiotosylate 4, and the *p*-toluenesulfinate anion served as the leaving group, which is why the *Z* geometry of the alkene remained unchanged. The thiolate anion can also be generated *in situ* from the corresponding thioacetate and sodium methoxide in methanol (entries 11–12). Such an approach is very convenient when a high-purity or stable thiol is not readily available. The developed method seems to be very versatile. The presence of additional functional groups including carbon–carbon multiple bonds (entries 10–12) and hydroxy (entry 2), ester (entry 3), azide (entry 4), amino (entry 5) aryl or heteroaryl (entries 6–9 and 15) moieties did not interfere with the formation of *Z*-alkenyl disulfanes 6. Arylthiol 5 – disulfane *Z*-6 exchange reaction was responsible for the formation of corresponding diaryl disulfane and moderate yield of 6f and 6m (entries 6 and 15). The exchange reaction can be limited by the excess of *Z*-4, what resulted in higher yield of 6f and 6m respectively (entries 7 and 16). l-Cysteine derivatives were also converted to the corresponding *Z*-alkenyl disulfanes 6k and 6l (entries 13–14). The biological activities of these compounds are expected to be higher than their *E*-isomer analogs.^43,44^

## Conclusions

We have developed the first simple and efficient diastereoselective method for the synthesis of functionalized unsymmetrical *Z*-alkenyl disulfanes under mild conditions in moderate to good yields. The developed method allows the preparation of unsymmetrical *Z*-alkenyl disulfanes bearing additional hydroxy, carboxy, or amino functionalities. Anti-fungal and anti-cancer activity studies of the *Z*-alkenyl l-cysteine disulfane derivatives are in progress.

## Experimental

### A typical procedure for the preparation of *Z*-alkenyl disulfanes 6 and representative analytical data

A compound *Z*-4 (0.67 mmol, 200 mg) was dissolved in dry DCM (3 mL) in the round bottom flask. Then a solution of thiol 5 (0.61 mmol) and NEt_3_ (0.61 mmol) in dry DCM (2 mL) was added. Reaction was stirred for 15 min. After this time solvent was evaporate and Et_2_O (10 mL) was added. Slurry was washed with water (10 mL) and aqueous phase was extracted 2 times with Et_2_O (2 × 10 mL). Organic layers were dried with MgSO_4_ and evaporated. The residue was purified by column chromatography (SiO_2_).

### (*Z*)-1-(dodec-1-yldisulfanyl)-oct-1-ene *Z*-6a

Chromatography, PE, *R*_f_ = 0.6; a colorless oil, yield 188 mg (90%) IR (ATR): 510 (s), 625 (m), 1490 (w), 2875 (m), 2900 (m) cm^−1^. ^1^H NMR (400 MHz, CDCl_3_) *δ* 6.09 (dt, *J* = 9.3, 1.4 Hz, 1H), 5.65 (dt, *J* = 9.3, 7.4 Hz, 1H), 2.72 (t, *J* = 7.3 Hz, 2H), 2.19 (qd, *J* = 7.4, 1.3 Hz, 2H), 1.70 (dt, *J* = 14.9, 7.2 Hz, 2H), 1.47–1.24 (m, 26H), 0.93–0.88 (m, 6H). ^13^C NMR (101 MHz, CDCl_3_) *δ* 132.66, 129.24, 39.07, 31.93, 31.67, 29.66, 29.64, 29.60, 29.51, 29.36, 29.23, 29.08, 28.91, 28.85, 28.80, 28.46, 22.70, 22.62, 14.13, 14.09. HRMS (ESI): *m*/*z* [M + H]^+^ calcd for C_20_H_41_S_2_: 345.2650; found: 345.2655.

### (*Z*)-11-(1-octen-1-yldisulfanyl)-undecan-1-ol *Z*-6b

Chromatography: PE:DCM 1:1; *R*_f_ = 0.4; a colorless oil, yield 173 mg (82%) IR (ATR): 755 (w), 1100 (w), 1500 (w), 1650 (w), 2875 (s), 2900 (s), 3300 (br) cm^−1^. ^1^H NMR (400 MHz, CDCl_3_) *δ* 6.09 (dt, *J* = 9.3, 1.3 Hz, 1H), 5.65 (dt, *J* = 9.3, 7.4 Hz, 1H), 3.66 (t, *J* = 6.6 Hz, 2H), 2.72 (t, *J* = 7.4 Hz, 2H), 2.19 (qd, *J* = 7.4, 1.3 Hz, 2H), 1.72–1.60 (m, 2H), 1.60–1.50 (m, 2H), 1.44–1.23 (m, 23H), 0.91 (t, *J* = 6.8 Hz, 3H). ^13^C NMR (101 MHz, CDCl_3_) *δ* 132.68, 129.22, 63.10, 39.06, 32.81, 31.67, 29.58, 29.51, 29.48, 29.42, 29.21, 29.07, 28.90, 28.84, 28.80, 28.44, 25.74, 22.62, 14.09. HRMS (ESI): *m*/*z* [M + H]^+^ calcd for C_19_H_39_OS_2_: 347.2442; found: 347.2438.

### (*Z*)-Methyl 11-(1-octen-1-yldisulfanyl)undecanoate *Z*-6c

Chromatography: PE:DCM 3:1; *R*_f_ = 0.4; a colorless oil, yield 201 mg (88%): IR (ATR): 500 (s), 625 (m), 1200 (w), 1490 (w), 1750 (m), 2875 (w), 2990 (m) cm^−1^. ^1^H NMR (400 MHz, CDCl_3_) *δ* 6.09 (dt, *J* = 9.3, 1.3 Hz, 1H), 5.65 (dt, *J* = 9.3, 7.4 Hz, 1H), 3.69 (s, 3H), 2.75–2.70 (m, 2H), 2.32 (t, *J* = 7.6 Hz, 2H), 2.19 (qd, *J* = 7.4, 1.3 Hz, 2H), 1.70–1.52 (m, 4H), 1.46–1.24 (m, 20H), 0.91 (t, *J* = 6.9 Hz, 3H). ^13^C NMR (101 MHz, CDCl_3_) *δ* 174.32, 132.67, 129.23, 51.45, 39.05, 34.11, 31.66, 29.43, 29.37, 29.23, 29.18, 29.14, 29.07, 28.89, 28.84, 28.79, 28.43, 24.95, 22.61, 14.09. HRMS (ESI): *m*/*z* [M + H]^+^ calcd for C_20_H_39_O_2_S_2_: 375.2391; found: 375.2396.

### (*Z*)-1-(11-azidoundec-1-yldisulfanyl)-oct-1-ene *Z*-6d

Chromatography: PE; *R*_f_ = 0.45; a colorless oil, yield 159 mg (70%) IR (ATR): 510 (s), 625 (m), 1260 (w), 1500 (w), 2110 (m), 2875 (m), 2900 (m)) cm^−1^. ^1^H NMR (400 MHz, CDCl_3_) *δ* 6.09 (dt, *J* = 9.3, 1.3 Hz, 1H), 5.65 (dt, *J* = 9.3, 7.4 Hz, 1H), 3.28 (t, *J* = 7.0 Hz, 2H), 2.73 (t, *J* = 7.4 Hz, 2H), 2.19 (qd, *J* = 7.4, 1.3 Hz, 2H), 1.75–1.55 (m, 4H), 1.47–1.24 (m, 22H), 0.91 (t, *J* = 6.9 Hz, 3H). ^13^C NMR (101 MHz, CDCl_3_) *δ* 132.68, 129.22, 51.50, 39.05, 31.67, 29.46, 29.20, 29.15, 29.07, 28.89, 28.85, 28.80, 28.43, 26.72, 22.62, 14.09. HRMS (ESI): *m*/*z* [M + H]^+^ calcd for C_19_H_38_N_3_S_2_: 372.2507; found: 372.2511.

### (*Z*)-1-(11-aminoundec-1-yldisulfanyl)-oct-1-ene *Z*-6e

Chromatography: DCM: MeOH 14:1; *R*_f_ = 0.3; a colorless oil, yield 162 mg (77%) IR (ATR): 510 (s), 625 (s), 800 (w), 1100 (w), 1490 (w), 1510 (w), 2875 (m), 2900 (s), 3500 (br) cm^−1^. ^1^H NMR (400 MHz, CDCl_3_) *δ* 6.09 (dt, *J* = 9.3, 1.3 Hz, 1H), 5.65 (dt, *J* = 9.3, 7.4 Hz, 1H), 2.87–2.81 (m, 2H), 2.75–2.70 (m, 2H), 2.18 (dt, *J* = 7.4, 4.2 Hz, 2H), 1.75–1.55 (m, 4H), 1.47–1.24 (m, 24H), 0.91 (t, *J* = 6.8 Hz, 3H). ^13^C NMR (101 MHz, CDCl_3_) *δ* 132.66, 129.25, 39.75, 39.07, 31.67, 29.50, 29.41, 29.25, 29.08, 28.92, 28.84, 28.48, 27.91, 26.53, 22.62, 14.11. HRMS (ESI): *m*/*z* [M + H]^+^ calcd for C_19_H_40_NS_2_: 346.2602; found: 346.2604.

### (*Z*)-1-(*p*-tolyldisulfanyl)-oct-1-ene *Z*-6f

Chromatography: PE; *R*_f_ = 0.8; a colorless oil, yield 65 mg (40%) IR (ATR): 500 (s), 625 (m), 780 (w), 1500 (w), 2875 (w), 2990 (m) cm^−1^. ^1^H NMR (400 MHz, CDCl_3_) *δ* 7.43 (d, *J* = 8.1 Hz, 2H), 7.15 (d, *J* = 8.1 Hz, 2H), 6.16 (dt, *J* = 9.3, 1.3 Hz, 1H), 5.75–5.65 (m, 1H), 2.36 (s, 3H), 2.19 (m, 2H), 1.46–1.25 (m, 8H), 0.91 (t, *J* = 6.9 Hz, 3H). ^13^C NMR (101 MHz, CDCl_3_) *δ* 137.52, 134.02, 129.74, 129.12, 127.92, 31.66, 29.08, 29.02, 28.89, 28.82, 22.61, 21.08, 14.09. HRMS (ESI): *m*/*z* [M + H]^+^ calcd for C_15_H_23_S_2_: 267.1241; found: 267.1243.

### (*Z*)-1-(furan-2-ylmethyldisulfanyl)-oct-1-ene *Z*-6g

Chromatography: PE; *R*_f_ = 0.7; a colorless oil, yield 111 mg (71%) IR (ATR): 600 (m), 740 (m), 770 (w), 900 (m), 1010 (m), 1125 (m), 1500 (w), 2875 (w), 2950 (m) cm^−1^. ^1^H NMR (400 MHz, CDCl_3_) *δ* 7.40 (dd, *J* = 1.8, 0.8 Hz, 1H), 6.34 (dd, *J* = 3.2, 1.8 Hz, 1H), 6.28 (d, *J* = 0.6 Hz, 1H), 5.84 (dt, *J* = 9.3, 1.3 Hz, 1H), 5.61 (dt, *J* = 9.3, 7.4 Hz, 1H), 3.94 (s, 2H), 2.20–2.12 (m, 2H), 1.42–1.15 (m, 8H), 0.91 (t, *J* = 6.7 Hz, 3H). ^13^C NMR (101 MHz, CDCl_3_) *δ* 150.16, 142.46, 133.11, 128.24, 110.64, 108.97, 35.72, 31.66, 29.05, 28.84, 28.76, 22.61, 14.08. HRMS (ESI): *m*/*z* [M + H]^+^ calcd for C_13_H_21_OS_2_: 257.1034; found: 257.1031.

### (*Z*)-1-(pyridin-4-yldisulfanyl)-oct-1-ene *Z*-6h

Chromatography: DCM; *R*_f_ = 0.4; a colorless oil, yield 80 mg (52%) IR (ATR): 510 (s), 625 (m), 740 (m), 770 (m), 1450 (w), 1490 (w), 1600 (s), 2875 (w), 2990 (w) cm^−1^. ^1^H NMR (400 MHz, CDCl_3_) *δ* 8.50 (d, *J* = 6.1 Hz, 2H), 7.41 (dd, *J* = 4.6, 1.6 Hz, 2H), 6.00 (dt, *J* = 9.2, 1.3 Hz, 1H), 5.81 (dt, *J* = 9.2, 7.5 Hz, 1H), 2.32 (qd, *J* = 7.4, 1.3 Hz, 2H), 1.52–1.23 (m, 8H), 0.93 (t, *J* = 6.9 Hz, 3H). ^13^C NMR (101 MHz, CDCl_3_) *δ* 149.50, 148.78, 135.62, 125.93, 120.14, 31.65, 29.09, 29.00, 28.89, 22.63, 14.09. HRMS (ESI): *m*/*z* [M + H]^+^ calcd for C_13_H_20_NS_2_: 254.1037; found: 254.1033.

### (*Z*)-1-(allyldisulfanyl)-oct-1-ene *Z*-6i

Chromatography: PE; *R*_f_ = 0.6; a colorless oil, yield 106 mg (80%) IR (ATR): 510 (s), 650 (s), 990 (w), 1510 (w), 2875 (w), 2990 (m) cm^−1^. ^1^H NMR (400 MHz, CDCl_3_) *δ* 6.08 (dt, *J* = 9.3, 1.3 Hz, 1H), 5.87 (ddt, *J* = 17.1, 10.0, 7.3 Hz, 1H), 5.66 (dt, *J* = 9.3, 7.4 Hz, 1H), 5.25–5.14 (m, 2H), 3.37 (dd, *J* = 7.3, 0.9 Hz, 2H), 2.19 (qd, *J* = 7.4, 1.3 Hz, 2H), 1.52–1.15 (m, 8H), 0.91 (t, *J* = 6.9 Hz, 3H). ^13^C NMR (101 MHz, CDCl_3_) *δ* 133.07, 132.99, 128.74, 118.66, 41.95, 31.66, 29.05, 28.85, 28.83, 22.61, 14.08. HRMS (ESI): *m*/*z* [M + H]^+^ calcd for C_11_H_21_S_2_: 217.1085; found: 217.1088.

### (*Z*)-1-(propargyldisulfanyl)-oct-1-ene *Z*-6j

Chromatography: PE; *R*_f_ = 0.55; a colorless oil, yield 102 mg (78%) IR (ATR): 625 (s), 1250 (w), 1500 (w), 2875 (w), 2990 (m), 3240 (w) cm^−1^. ^1^H NMR (400 MHz, CDCl_3_) *δ* 6.21 (dt, *J* = 9.3, 1.4 Hz, 1H), 5.73 (dt, *J* = 9.3, 7.4 Hz, 1H), 3.49 (d, *J* = 2.6 Hz, 2H), 2.32 (t, *J* = 2.6 Hz, 1H), 2.21 (qd, *J* = 7.4, 1.3 Hz, 2H), 1.46–1.25 (m, 8H), 0.91 (t, *J* = 6.9 Hz, 3H). ^13^C NMR (101 MHz, CDCl_3_) *δ* 134.23, 127.62, 79.33, 72.41, 31.64, 29.04, 28.82, 28.76, 27.14, 22.61, 14.09. HRMS (ESI): *m*/*z* [M + H]^+^ calcd for C_11_H_19_S_2_: 215.0928; found: 215.0932.

### (*Z*)-Ethyl (*R*)-2-amino-3-(oct-1-ene-1-yldisulfanyl)propanoate *Z*-6k

Chromatography: DCM: MeOH 14:1; *R*_f_ = 0.35; a colorless oil, yield 107 mg (60%) IR (ATR): 510 (s), 625 (m), 1010 (w), 1200 (w), 1500 (w), 1750 (m), 2875 (w), 2990 (w), 3500 (br) cm^−1^. ^1^H NMR (400 MHz, CDCl_3_) *δ* 6.11 (dt, *J* = 9.3, 1.3 Hz, 1H), 5.70 (dq, *J* = 8.9, 7.5 Hz, 1H), 4.22 (q, *J* = 7.1 Hz, 2H), 3.81 (dd, *J* = 8.0, 4.5 Hz, 1H), 3.15 (dd, *J* = 13.7, 4.5 Hz, 1H), 2.89 (dd, *J* = 13.7, 8.0 Hz, 1H), 2.18 (qd, *J* = 7.4, 1.3 Hz, 2H), 1.57–1.07 (m, 8H), 0.89 (t, *J* = 6.8 Hz, 3H). ^13^C NMR (101 MHz, CDCl_3_) *δ* 173.66, 134.02, 127.86, 61.35, 53.43, 43.90, 31.63, 29.01, 28.85, 28.80, 22.58, 14.18, 14.07. HRMS (ESI): *m*/*z* [M + H]^+^ calcd for C_13_H_26_NO_2_S_2_: 292.1399; found: 292.1403.

### (*Z*)-Ethyl (*R*)-2-((*tert*-butoxycarbonyl)amino)-3-(oct-1-ene-1-yldisulfanyl)propanoate *Z*-6l

Chromatography: PE: DCM 1:2; *R*_f_ = 0.5; a colorless oil, yield 141 mg (59%) IR (ATR): 500 (s), 625 (m), 1010 (m), 1200 (s), 1375 (m), 1625 (m), 1750 (m), 2875 (w), 2990 (w), 3000 (w) cm^−1^. ^1^H NMR (400 MHz, CDCl_3_) *δ* 6.11 (dt, *J* = 9.3, 1.3 Hz, 1H), 5.72 (dt, *J* = 9.3, 7.4 Hz, 1H), 5.35 (d, *J* = 7.3 Hz, 1H), 4.61 (d, *J* = 6.1 Hz, 1H), 4.28–4.21 (m, 2H), 3.19 (ddd, *J* = 19.8, 14.0, 5.2 Hz, 2H), 2.17 (qd, *J* = 7.4, 1.2 Hz, 2H), 1.48 (s, 9H), 1.44–1.25 (m, 11H), 0.90 (t, *J* = 6.9 Hz, 3H). ^13^C NMR (101 MHz, CDCl_3_) *δ* 170.70, 155.06, 133.93, 127.97, 80.14, 61.79, 53.43, 53.07, 41.53, 31.64, 29.04, 28.86, 28.85, 28.31, 22.59, 14.13, 14.08. HRMS (ESI): *m*/*z* [M + H]^+^ calcd for C_18_H_34_NO_4_S_2_: 392.1929; found: 392.1934.

### (*Z*)-1-(4-methoxylphenyldisulfanyl)-oct-1-ene *Z*-6m

Chromatography: PE; *R*_f_ = 0.3; a colorless oil, yield 74 mg (43%) IR (ATR): 523 (w), 823 (m), 1031 (m), 1244 (s), 1490 (s), 1589 (m), 2853 (s), 2923 (s), 3333 (br) cm^−1^. ^1^H NMR (400 MHz, CDCl_3_) *δ* 7.52–7.47 (m, 2H), 6.91–6.83 (m, 2H), 6.22 (dt, *J* = 9.3, 1.3 Hz, 1H), 5.71 (dt, *J* = 9.2, 7.4 Hz, 1H), 3.83 (s, 3H), 2.19–2.07 (m, 2H), 1.44–1.17 (m, 8H), 0.90 (t, *J* = 6.9 Hz, 3H). ^13^C NMR (101 MHz, CDCl_3_) *δ* 159.86, 134.14, 132.49, 128.34, 127.90, 114.61, 55.38, 31.64, 29.00, 28.83, 28.80, 22.59, 14.09. HRMS (ESI): *m*/*z* [M + H]^+^ calcd for C_15_H_23_OS_2_: 283.4725; found: 283.4729.

## Conflicts of interest

There are no conflicts to declare.

## Supplementary Material

RA-008-C8RA00659H-s001
