# Supplementary material for: Convenient and efficient synthesis of functionalized unsymmetrical Z-alkenyl disulfanes
Source: RSC Adv. 2018 Mar 7;8(18):9718–22. doi: 10.1039/c8ra00659h (PMC9078699; doi:10.1039/c8ra00659h)
Supplement: RA-008-C8RA00659H-s001 [file RA-008-C8RA00659H-s001.pdf]

Electronic Supplementary Material (ESI) for RSC Advances.
This journal is © The Royal Society of Chemistry 2018

Convenient and efficient synthesis of functionalized unsymmetrical *Z*-alkenyl disulfanes

Supporting Information

Mateusz Musiejuk, Justyna Doroszuk and Dariusz Witt*

Department of Organic Chemistry, Faculty of Chemistry, Gdansk University of Technology, Narutowicza 11/12, 80-233 Gdansk, Poland

Fax +48(58)3472694; E-mail: chemwitt@pg.gda.pl

Content

1	General information	1
2	Typical procedure for the preparation of sodium 4-methylbenzenesulfonotioate.	2
3	General procedure of synthesis (*E*)-1-octene-1-ylphenyliodinium tetrafluoroborate *E*-3	2
4	General procedure of synthesis (*Z*)-1-(4-toluenethiosulfonyl)-1-octene *Z*-4	3
5	Typical procedure of synthesis unsymmetrical vinyl disulfanes *Z*-6.	4

1. General information.

All thiols **5** were purchased from ProChimia (www.prochimia.com), sodium 4-toluenesulfonothioate was obtained from sodium 4-toluenesulfinate purchased from Merck. Allyl bromide, propargyl bromide, catecholborane, dibromoborane in complex with dimethyl sulfide, and diacethoxyiodobenzene were purchased from Merck. Dry methanol and dichloromethane were obtained by standard procedure. TLC was performed with silica gel Polygram SIL G/UV254. Column chromatography was performed using silica gel 60 (230-400 mesh, Merck). NMR spectra were recorded on Brucker 400 MHz spectrometer. The residual solvent peak was used as the internal reference ($CDCl_3$: δ=7.26 ppm for ^1H, δ=77.0 ppm for ^{13}C). IR spectra were recorded on Nicolet Is50 FT-IR spectrometer by ATR method. Melting points were measured with a Gallenkamp 7936B apparatus. ESI-MS spectra were recorded on a Mariner PerSeptive Biosystem.

2. **Typical procedure for the preparation of sodium 4-methylbenzenesulfonotioate.**

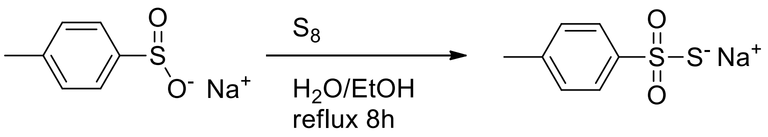

To a stirred solution of sodium 4-methylbenzenesulfinate (112 mmol) in H_2O/EtOH (100 mL/ 100 mL) was added sulfur (122 mmol). A mixture was heated under reflux for 8 hours. After cooling to rt excess of sulfur was filtered off and the solution was evaporated under diminished pressure. The wet solid was dried under vacuum for 9 hours at room temperature to provide 18.8g (80%) of sodium 4-methylbenzenesulfonotioate.

3. **General procedure of synthesis (*E*)-1-octene-1-ylphenyliodinium tetrafluoroborate *E*-3**

a. Synthesis of (*E*)-1-octene-1-ylboronic acid *E*-**2** – Method A

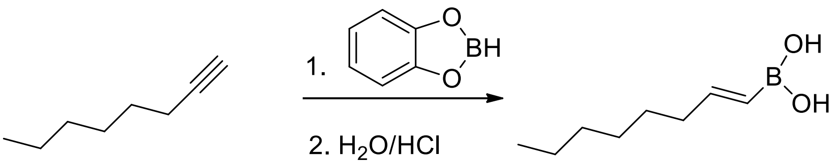

1 *E*-**2**

To dry 2-necked round bottom flask with condenser and septum 1-oktyne (1 eq. 10.1 mmol, 1.12g) was added, then catecholborane (1 eq., 1M in THF, 10.1 mL) was added by syringe. The reaction mixture was refluxed for 1h under N_2. After this time reaction mixture was evaporated. The crude catecholboronic acid ester was hydrolyzed using water with few drops of conc. HCl. The mixture was shaken for 30 min and filtered. The crude product was recrystallized from water to provide (*E*)-1-octene-1-ylboronic acid 0.63 g in 40% yield as a white powder mp 83-85 °C.

b. Synthesis of (*E*)-1-octene-1-ylboronic acid *E*-**2** – Method B

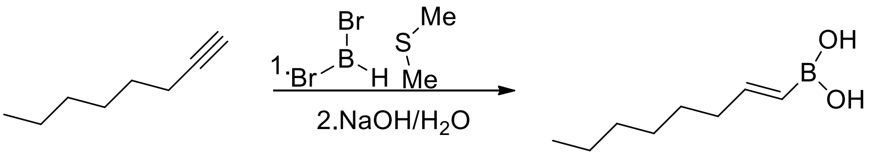

1 *E*-**2**

A solution of 1-oktyne (1.1 eq. 44 mmol, 4.84g) in dry DCM (20 mL) in dry round bottom flask was cooled to 5 °C under N_2. Then solution of dibromoborane complex with dimethyl sulfide (1 eq. 40 mmol, 1M in DCM, 40 mL) was added dropwise for 1h. A mixture was stirred for 4h at 5-10 °C. After this time reaction mixture was cooled to 0 °C and the solution of NaOH (2 eq. 80 mmol, 1.6 g) in water (20 mL) was added at 0 °C. Mixture was stirred for 2h at 0 °C, then water (50 mL) was added and precipitate was filtered off, washed with cold water, and dried under vacuum to provide 5.0 g of (*E*)-1-octene-1-ylboronic acid in 80% yield as a white powder mp 83-85 °C.

c. Synthesis of (*E*)-1-octene-1-ylphenyliodinium tetrafluoroborate *E*-**3**

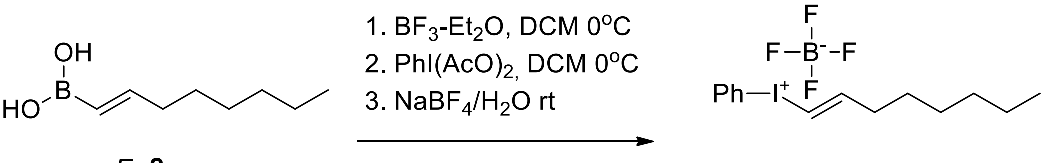

E- **2**

E- **3**

To dry 2-necked round bottom flask with thermometer and septum (*E*)-1-octene-1-ylboronic acid (1 eq. 1mmol, 156mg) and dry DCM (2 mL) were added. A slurry was cooled to 0 °C and BF_3 Et_2O (1.2 eq., 1.2 mmol, 0.15 mL) was added. A solution was stirred for 15min, and solution of diacethoxyiodobenzene (1.2eq, 1.2 mmol, 387mg) in DCM (1 mL) was added at 0 °C. Mixture was stirred for 1h at 0 °C and saturated aqueous solution of $NaBF_4$ (5 mL) was added with vigorous stirring. After 15 min mixture was extracted 3 times with DCM (3x20 mL). Organic phases were dried $MgSO_4$ and evaporated. A crude product was washed 5 times with petroleum ether (5x 10mL), and mixture PE/Et_2O (1/1) (3x 10 mL) to provide 0.26 g of (*E*)-1-octene-1-ylphenyliodinium tetrafluoroborate *E*-**3** as brownish oil in 65% yield. Compound should be stored in the freezer.

4. General procedure of synthesis (*Z*)-1-(4-toluenethiosulfonyl)-1-octene *Z*-4

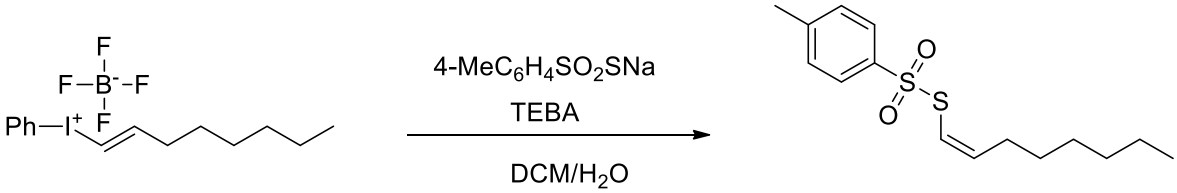

E- **3**

Z- **4**

A solution of compound *E*-**3** (1eq., 5 mmol, 2.0 g) in DCM (5 mL) was cooled to 5 – 0 °C and solution of sodium 4-methylbenzenesulfonotioate (1eq., 5 mmol, 1.05g) and triethylbenzylammonium chloride TEBA (0.1eq., 0.5 mmol, 114mg) in water (5 mL) was added. The reaction mixture was stirred for 2.5 h at 5 °C. Then organic phase was collected, and aqueous phase was extracted with DCM (3x 10 mL). Organic layers were dried with $MgSO_4$ and evaporated. Crude compound was purified by column chromatography (SiO_2) using PE/DCM (1:1) as eluent to give 1.19 g of (*Z*)-1-(4-toluenethiosulfonyl)-1-octene Z-**4** as a colorless oil in 80% yield.

5. **Typical procedure of synthesis unsymmetrical vinyl disulfanes *Z*-6.**
    a. From thiol

Compound Z-**4** (1.1eq, 0.67 mmol, 200mg) was dissolved in dry DCM (3 mL) in the round bottom flask. Then a solution of thiol **5** (1eq, 0.61 mmol) and NEt_3 (1eq, 0.61 mmol) in dry DCM (2 mL) was added. Reaction was stirred for 15 min. After this time solvent was evaporate and Et_2O (10 mL) was added. Slurry was washed with water (10 mL) and aqueous phase was extracted 2 times with Et_2O (2x 10 mL). Organic layers were dried with $MgSO_4$ and evaporated. Crude compounds were purified using column chromatography.

    b. From *S*-Acetylthiol **5i** or **5j**

Compound **5i** or **5j** (1 eq, 0.61 mmol) was added to a solution of MeONa (1 eq, 0.61 mmol) in dry MeOH (2 mL). Then a solution of thiotosylate *Z*-**4** (1.1.eq, 0.67 mmol, 200 mg) in dry MeOH (3 mL) was added. The reaction mixture was stirred for 15 min. After this time solvent was evaporated and the residue was purified by column chromatography (SiO_2).

6. **Spectral characterization of compound *E*-2, *E*-3, *Z*-4**

(*E*)-1-octene-1-ylboronic acid *E*-**2**

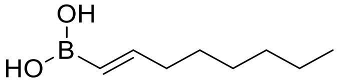

A white solid, yield 5.0g (80%) mp 83-85 °C (lit. 77-83 °C)

^{1}H NMR (400 MHz, CD_3OD) δ 6.54 (dt, *J* = 17.9, 6.4 Hz, 1H), 5.43 (dt, *J* = 17.9, 1.5 Hz, 1H), 2.30-2.12 (m, 2H), 1.60-1.2 (m, 8H), 0.90 (t, *J* = 6.9, 3H).

I. J. S. Fairlamb, L. R. Marrison, J. M. Dickinson,F.-J. Lua, J. P. Schmidt, *Bioorg. Med. Chem.*, 2004, **12**, 4285.

(*E*)-1-octene-1-yl(phenyl)iodonium tetrafluoroborate *E*-**3**

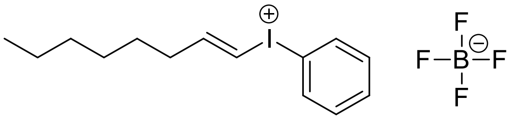

A colorless oil; yield 0.26g (65%)

^{1}H NMR (400 MHz, $CDCl_3$) δ 7.91 (dd, *J* = 5.5 Hz, 0.6 Hz, 2H), 7.64 (t, *J* = 6.1 Hz, 1H), 7.47 (td, *J* = 5.0 Hz, 1.3 Hz, 2H), 6.29-6.86 (m, 1H), 6.69 (d, *J* = 10.9 Hz, 1H), 2.32 (q, *J* = 5.7 Hz, 2H), 1.29-0.99 (m, 8H), 0.78 (t, *J* = 6.0 Hz, 3H)

M. Ochiai, M. Toyonari, T. Nagaoka, D.-W. Chen and M. Kida, *Tetrahedron Lett.*, 1997, **38**, 6709.

(*Z*)-*S*-oktene-1-yl 4-toluenethiosulfonate *Z*-**4**

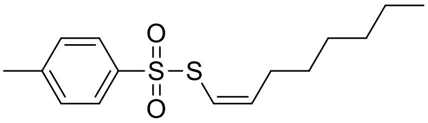

Chromatography: PE:DCM 4:1 a colorless oil, yield 1.19 g (80%)

IR (ATR): 500 (m), 625 (s), 700 (m), 800 (m), 1100 (s), 1380 (s), 1600 (w), 2800 (w), 2990 (w) cm^{-1}.

^{1}H NMR (400 MHz, $CDCl_3$) δ 7.80 (d, *J* = 8.1 Hz, 2H), 7.34 (d, *J* = 8.1 Hz, 2H), 6.25 (dt, *J* = 8.8, 1.1 Hz, 1H), 6.18 (dt, *J* = 8.8, 7.2 Hz, 1H), 2.46 (s, 3H), 1.99 (t, *J* = 6.7 Hz, 2H), 1.30-1.08 (m, 8H), 0.88 (t, *J* = 7.1 Hz, 3H).

^{13}C NMR (101 MHz, $CDCl_3$) δ 145.13, 144.61, 141.57, 129.61, 127.29, 115.90, 31.51, 29.26, 28.66, 28.40, 22.48, 21.64, 14.04.

HRMS (ESI): m/z $[M + H]^+$calcd for $C_{15}H_{23}O_2S_2$: 299.1139; found: 299.1145.

7. Copy of IR, ^{1}H NMR and ^{13}C NMR spectra for compounds *Z*-**4** and *Z*-**6**

IR (ATR):***Z*-4**

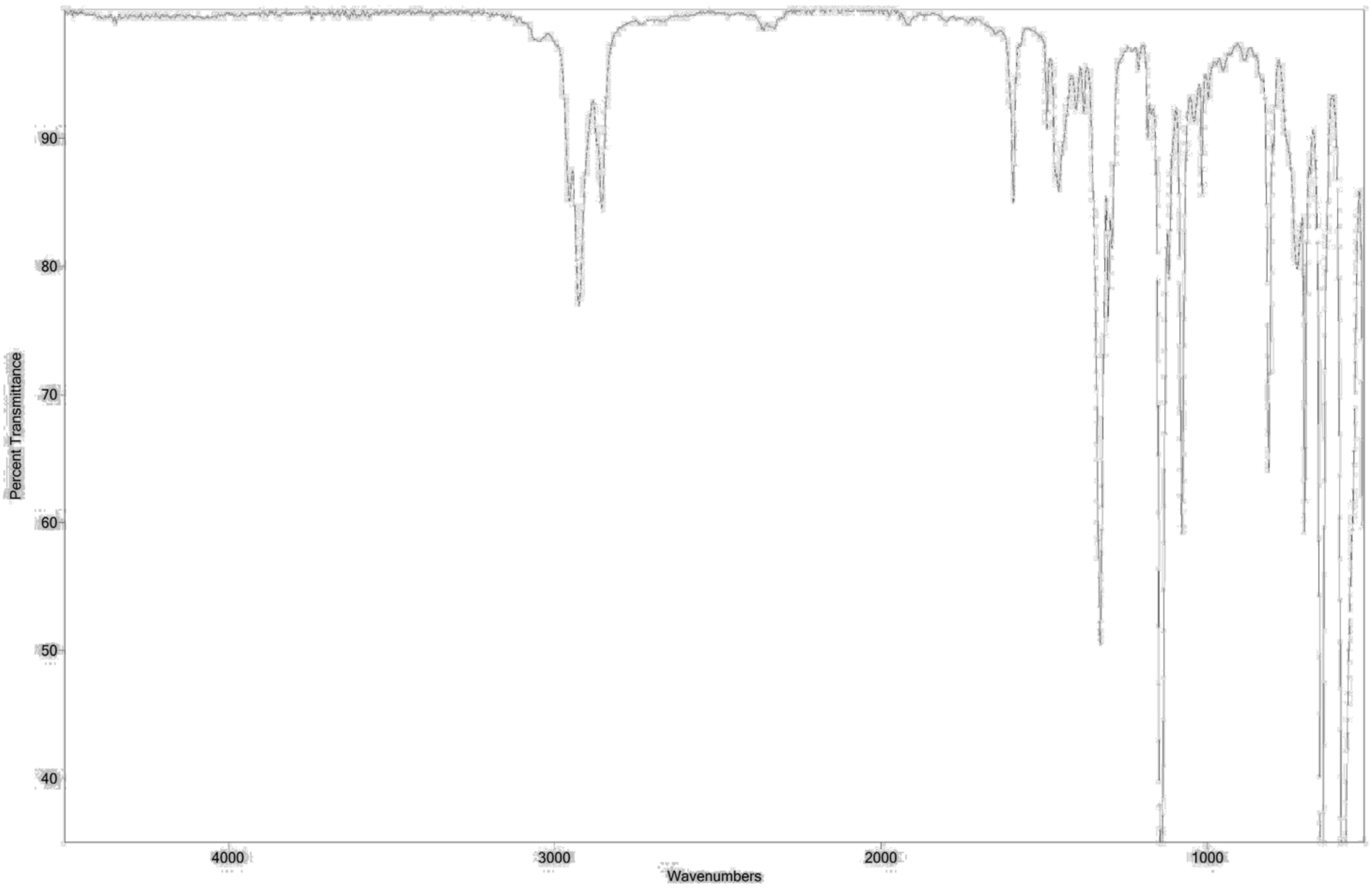

Percent Transmittance
90
80
70
60
50
40
4000
3000
2000
1000
Wavenumbers

Z-4

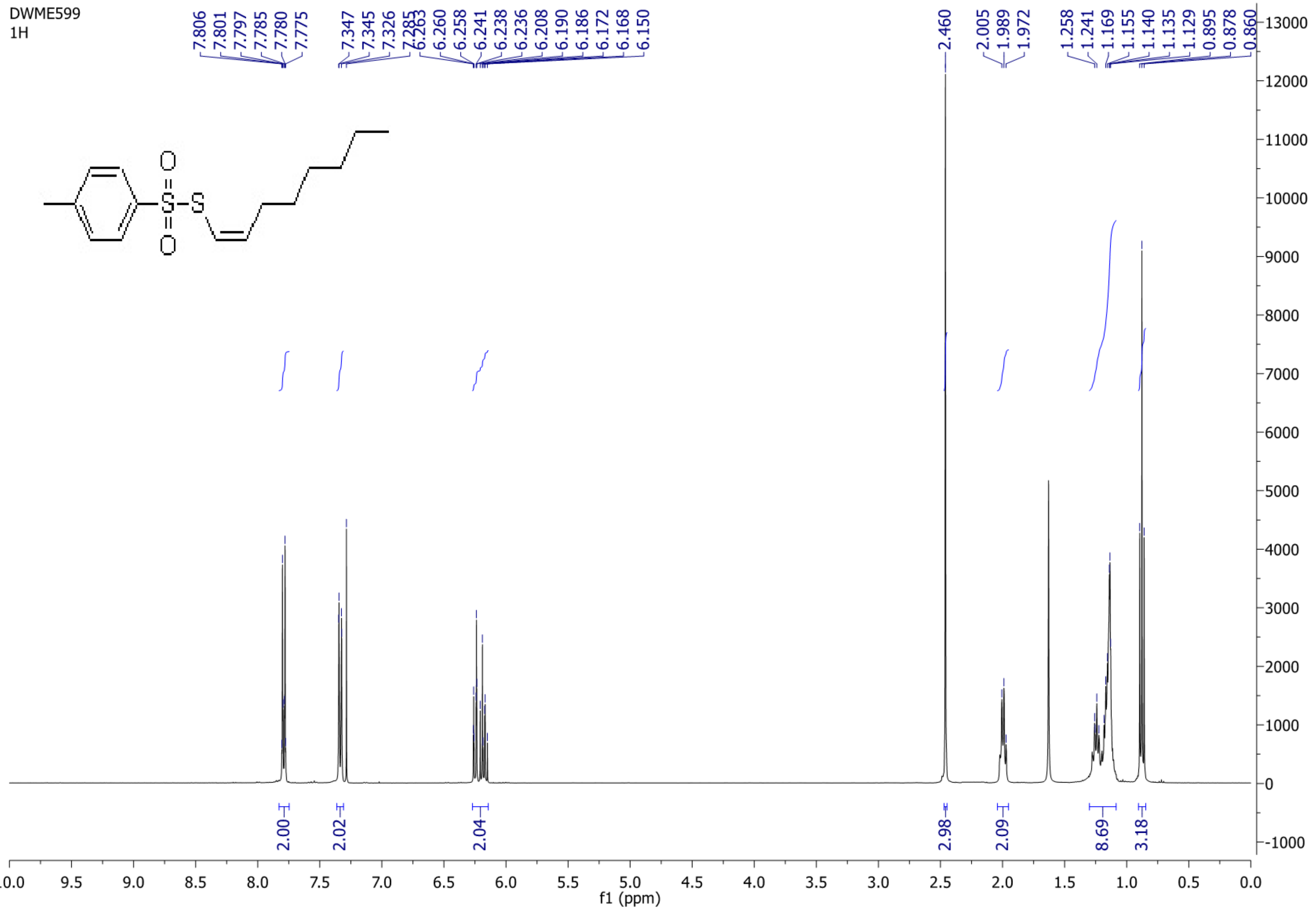
DWME599
1H
7.806
7.801
7.797
7.785
7.780
7.775
7.347
7.345
7.326
7.285
6.263
6.260
6.258
6.241
6.238
6.236
6.208
6.190
6.186
6.172
6.168
6.150
2.460
2.005
1.989
1.972
1.258
1.241
1.169
1.155
1.140
1.135
1.129
0.895
0.878
0.860
2.00
2.02
2.04
2.98
2.09
8.69
3.18
f1 (ppm)

***Z*-4**

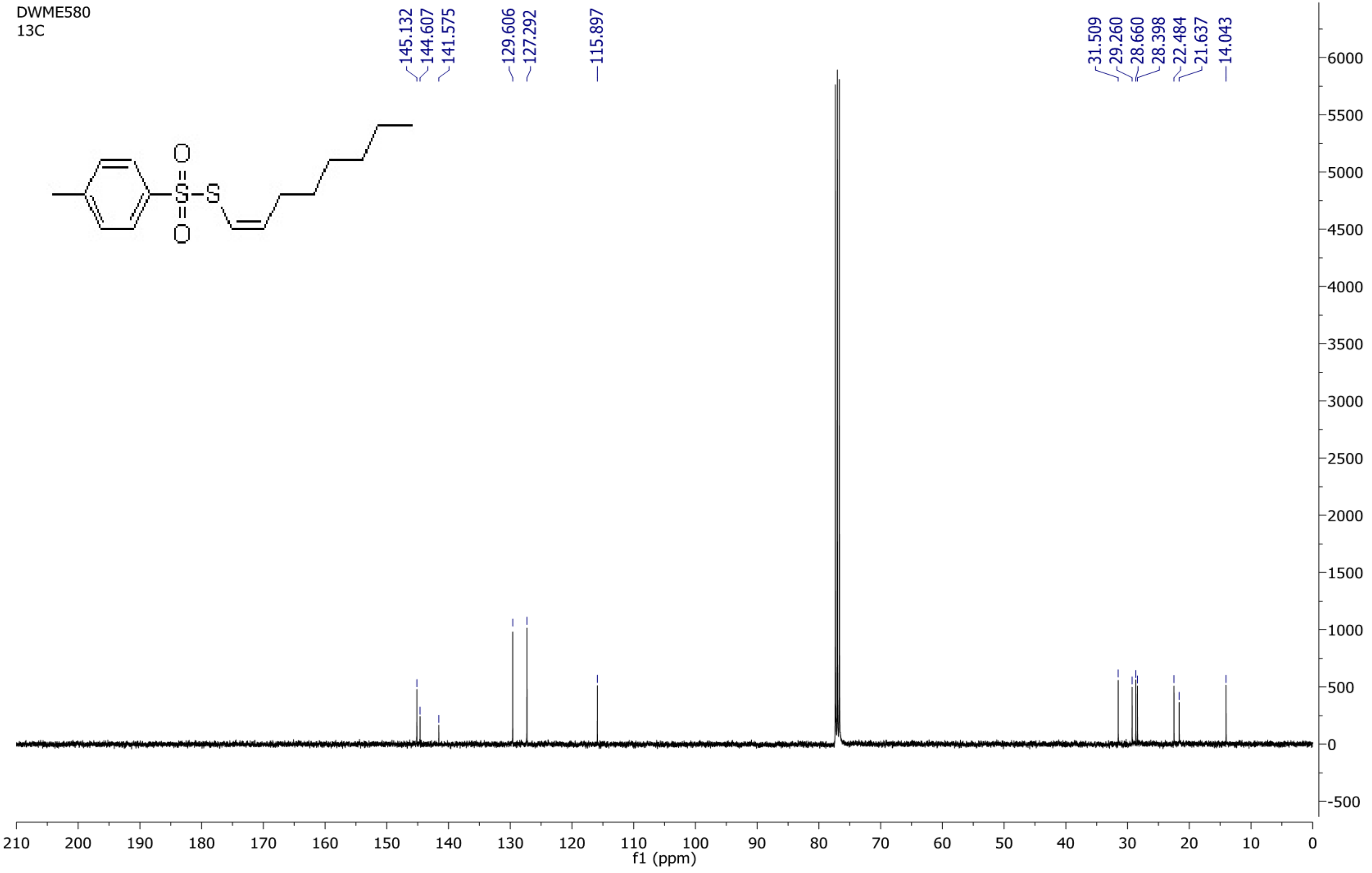

DWME580
13C
145.132
144.607
141.575
129.606
127.292
115.897
31.509
29.260
28.660
28.398
22.484
21.637
14.043
6000
5500
5000
4500
4000
3500
3000
2500
2000
1500
1000
500
0
-500
210
200
190
180
170
160
150
140
130
120
110
100
90
80
70
60
50
40
30
20
10
0
f1 (ppm)

IR (ATR): **Z-6a**

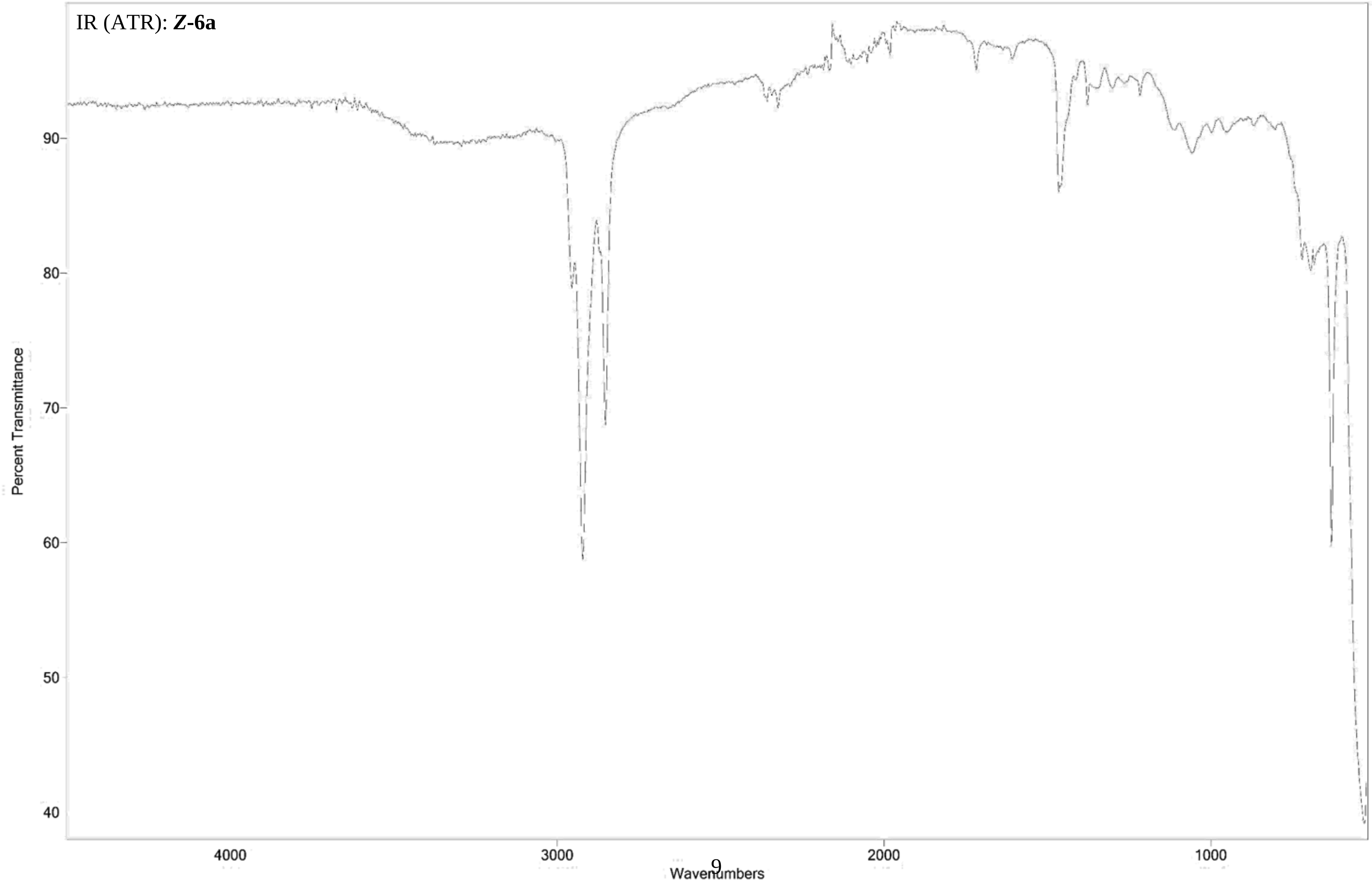

Percent Transmittance
90
80
70
60
50
40
4000
3000
2000
1000
Wavenumbers

Z-6a

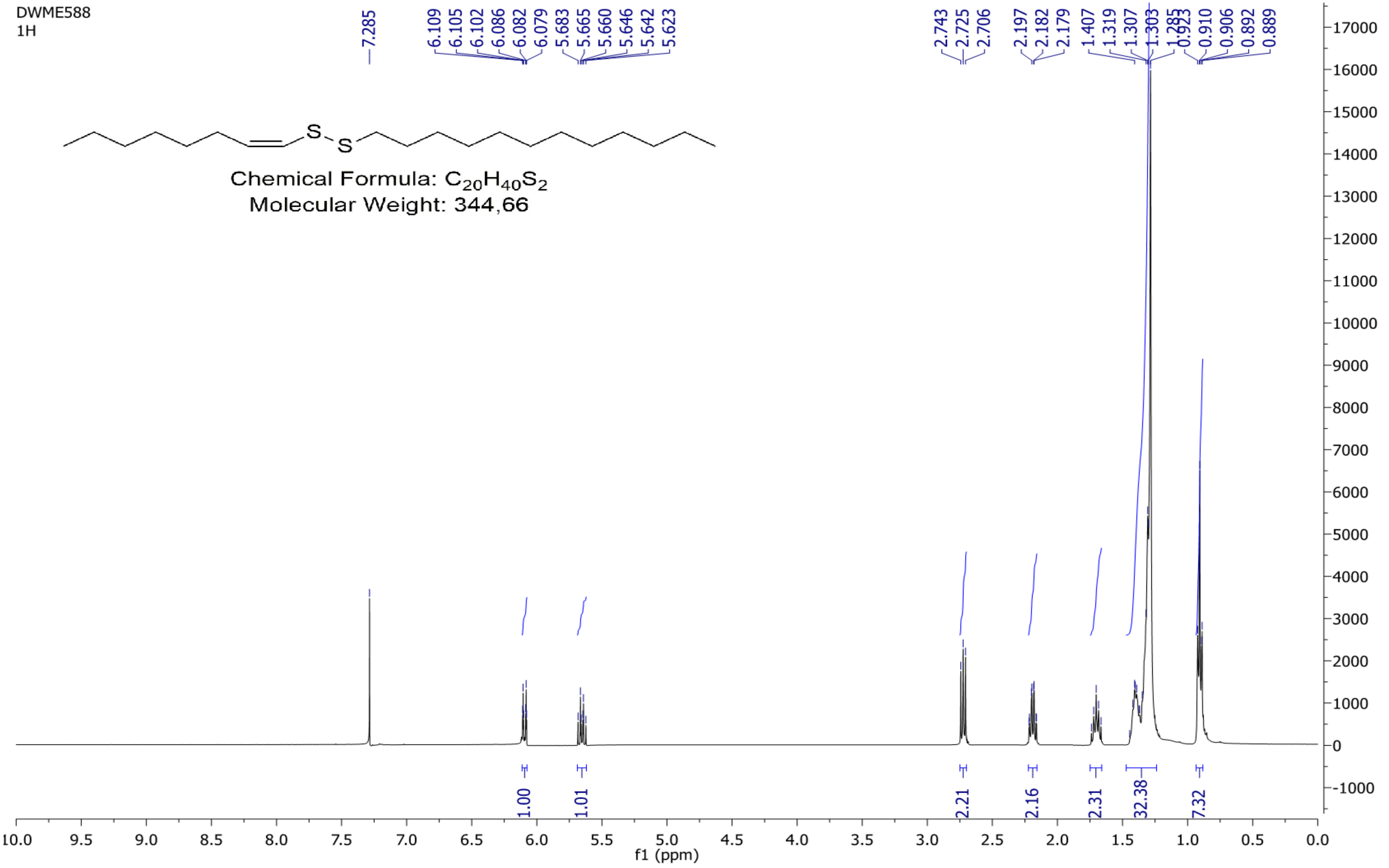
DWME588
1H
7.285
6.109
6.105
6.102
6.086
6.082
6.079
5.683
5.665
5.660
5.646
5.642
5.623
2.743
2.725
2.706
2.197
2.182
2.179
1.407
1.319
1.307
1.303
1.285
0.923
0.910
0.906
0.892
0.889
Chemical Formula: $C_{20}H_{40}S_2$
Molecular Weight: 344,66
1.00
1.01
2.21
2.16
2.31
32.38
7.32
f1 (ppm)

Z-6a

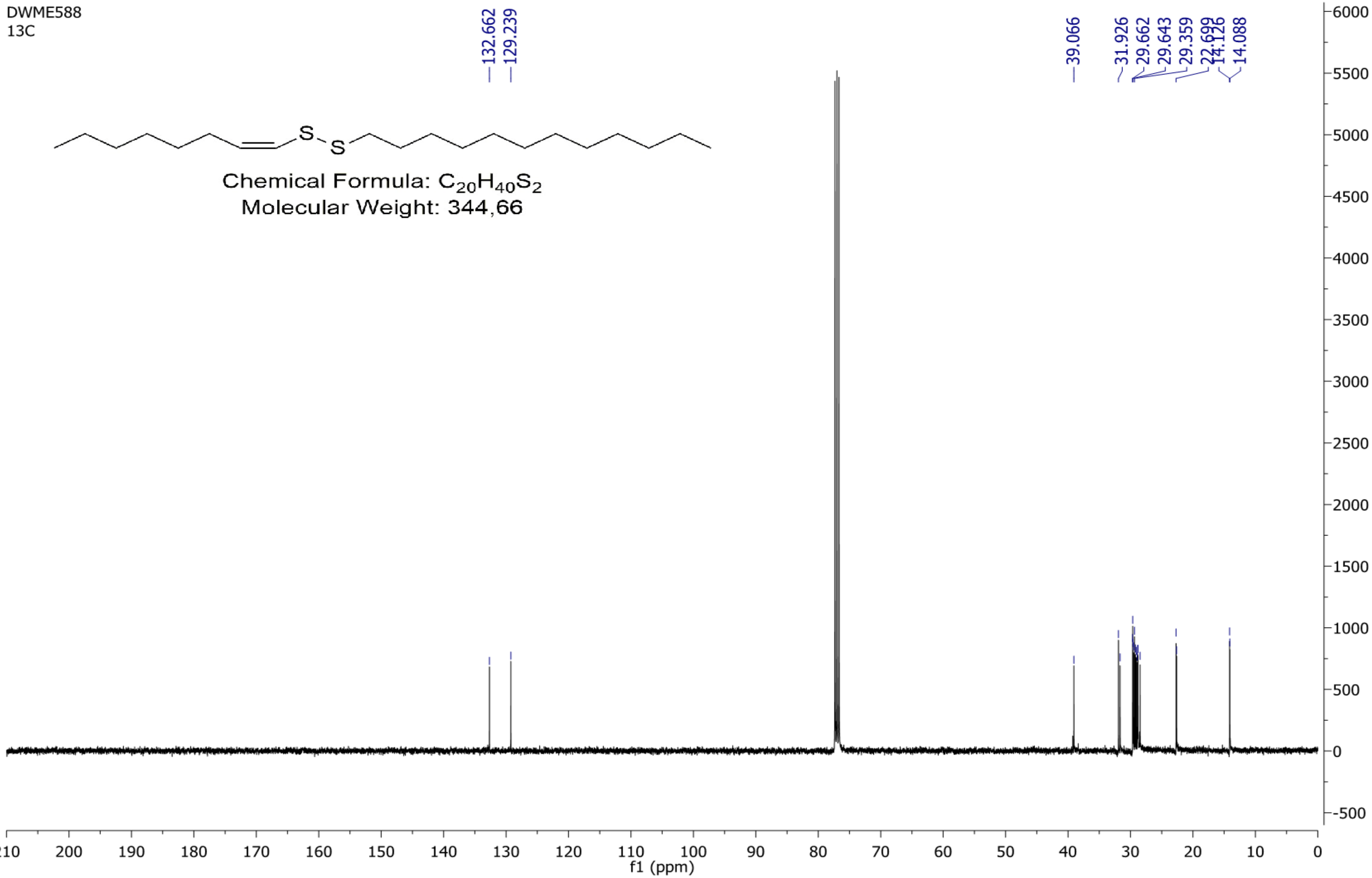
DWME588
13C
132.662
129.239
39.066
31.926
29.662
29.643
29.359
22.699
14.126
14.088
Chemical Formula: $C_{20}H_{40}S_2$
Molecular Weight: 344,66
S
S
6000
5500
5000
4500
4000
3500
3000
2500
2000
1500
1000
500
0
-500
210
200
190
180
170
160
150
140
130
120
110
100
90
80
70
60
50
40
30
20
10
0
f1 (ppm)

IR (ATR) ***Z*-6b**

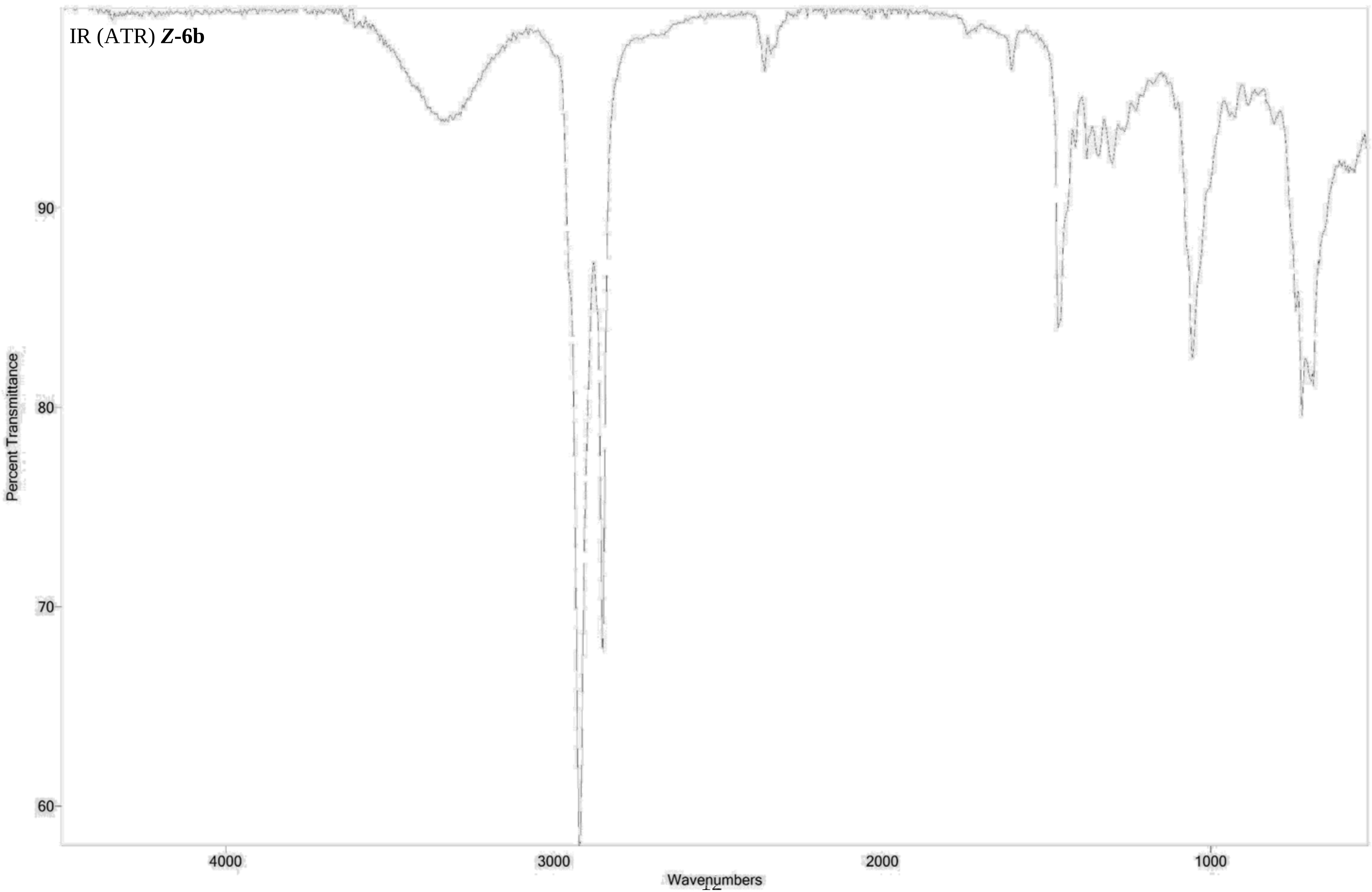
90
80
70
60
Percent Transmittance
4000
3000
2000
1000
Wavenumbers

Z-6b

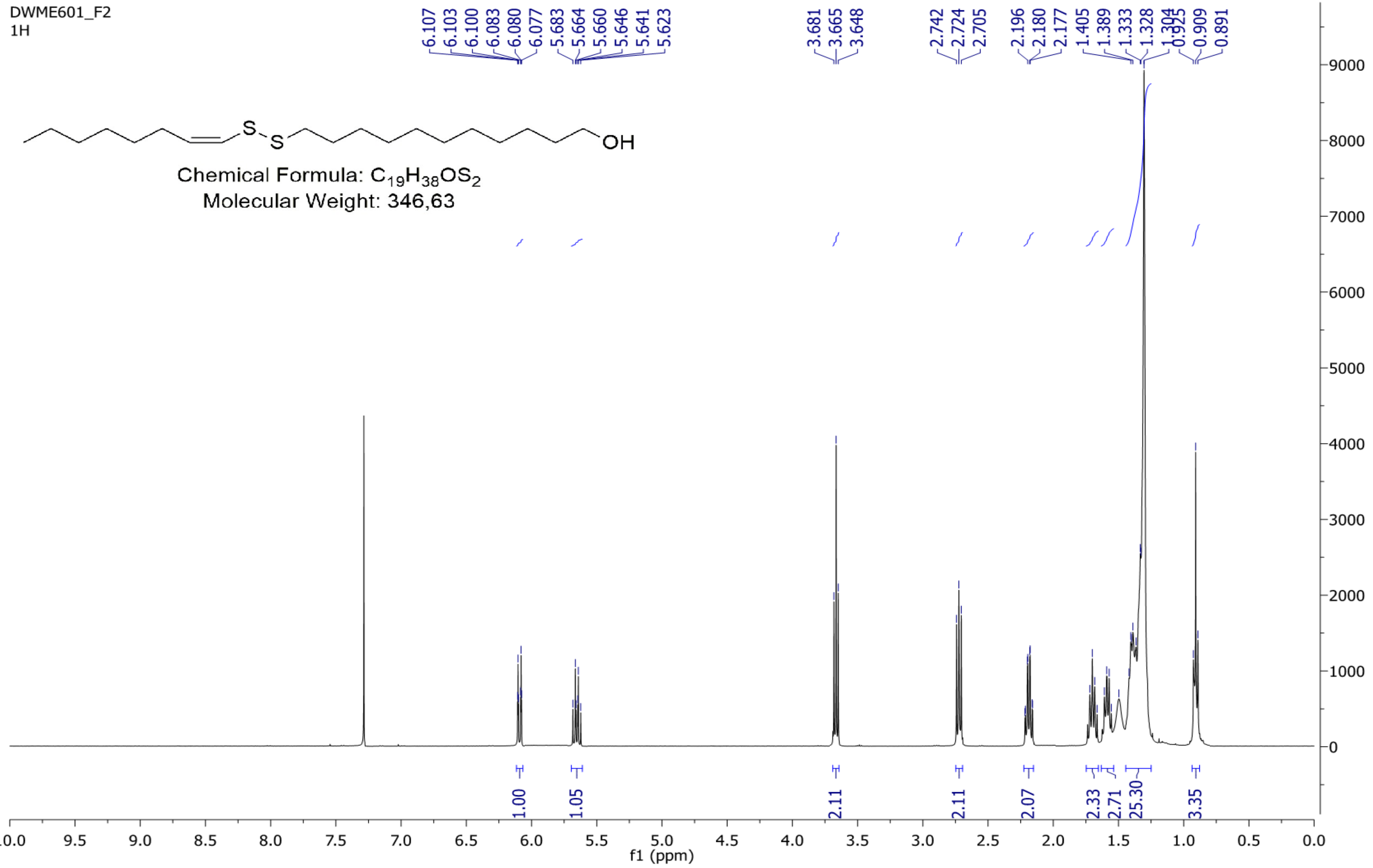
DWME601_F2
1H
6.107 6.103 6.100 6.083 6.080 6.077 5.683 5.664 5.660 5.646 5.641 5.623
3.681 3.665 3.648
2.742 2.724 2.705
2.196 2.180 2.177
1.405 1.389 1.333 1.328 1.304 0.925 0.909 0.891
S
S
OH
Chemical Formula: $C_{19}H_{38}OS_2$
Molecular Weight: 346,63
1.00 1.05 2.11 2.11 2.07 2.33 2.71 25.30 3.35
10.0 9.5 9.0 8.5 8.0 7.5 7.0 6.5 6.0 5.5 5.0 4.5 4.0 3.5 3.0 2.5 2.0 1.5 1.0 0.5 0.0
f1 (ppm)
9000 8000 7000 6000 5000 4000 3000 2000 1000 0

Z-6b

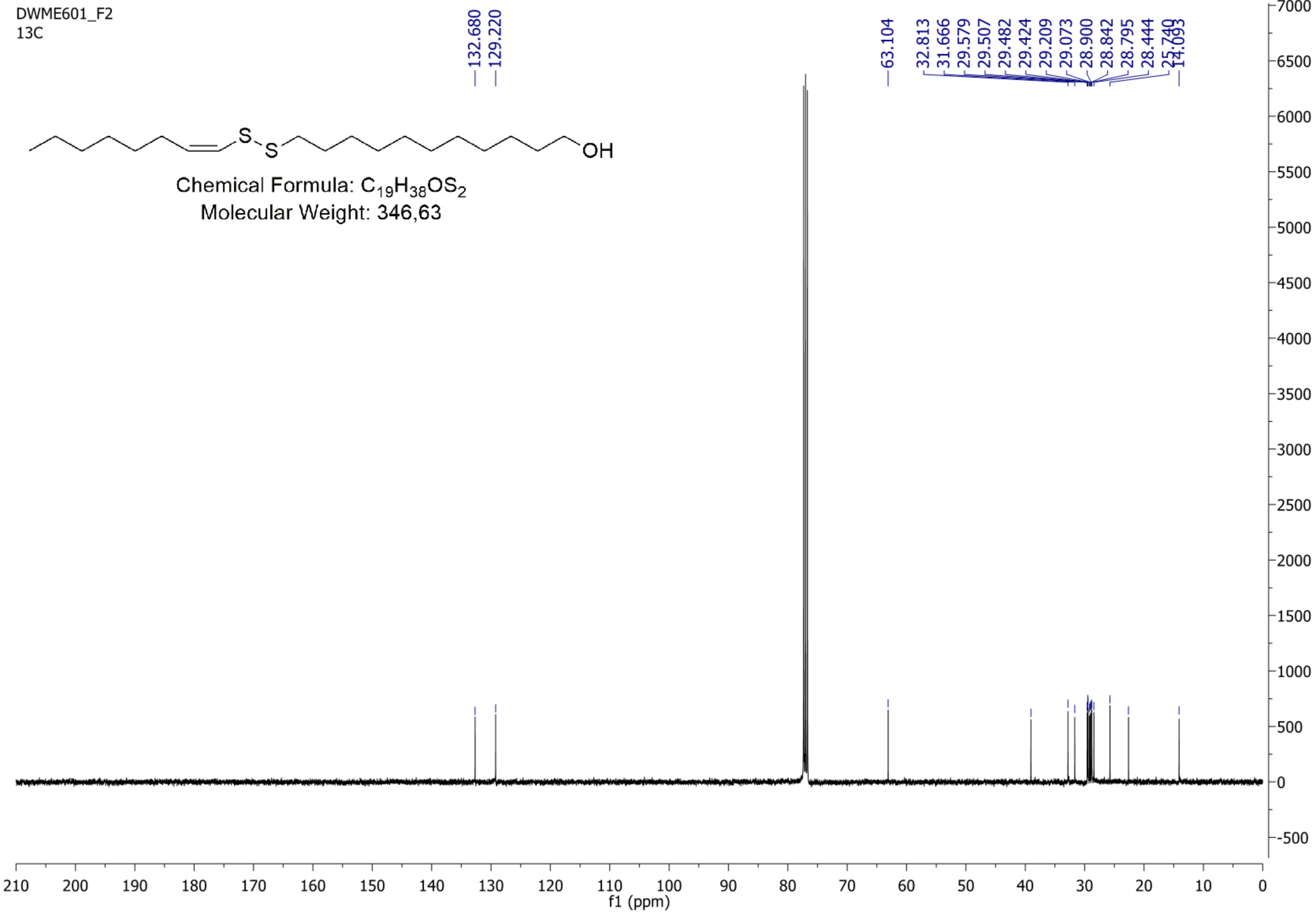

DWME601_F2
13C
132.680
129.220
63.104
32.813
31.666
29.579
29.507
29.482
29.424
29.209
29.073
28.900
28.842
28.795
28.444
14.093
OH
S
S
Chemical Formula: $C_{19}H_{38}OS_2$
Molecular Weight: 346,63
f1 (ppm)

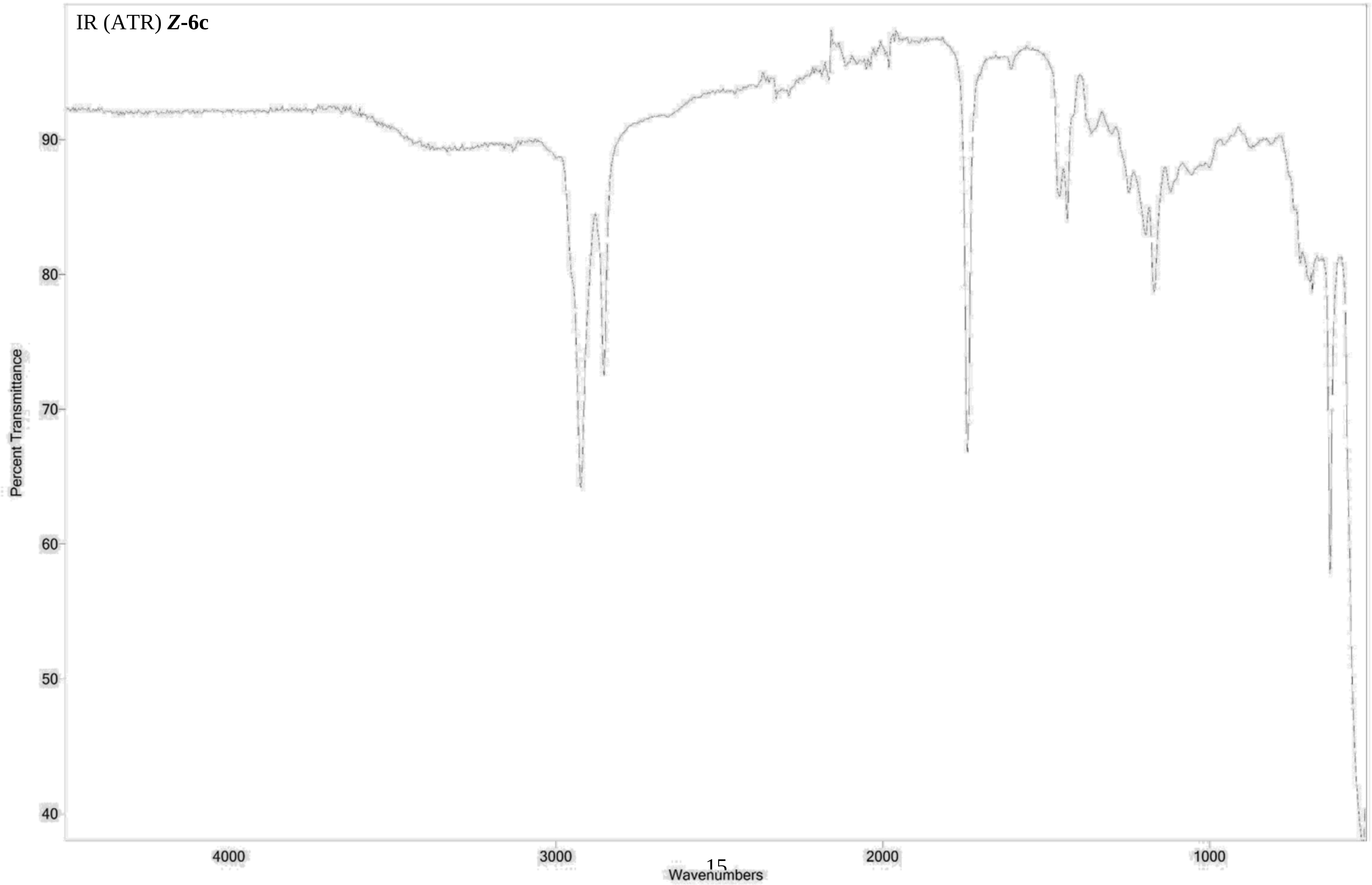

IR (ATR) ***Z*-6c**
Percent Transmittance
90
80
70
60
50
40
4000
3000
2000
1000
Wavenumbers

Z-6c

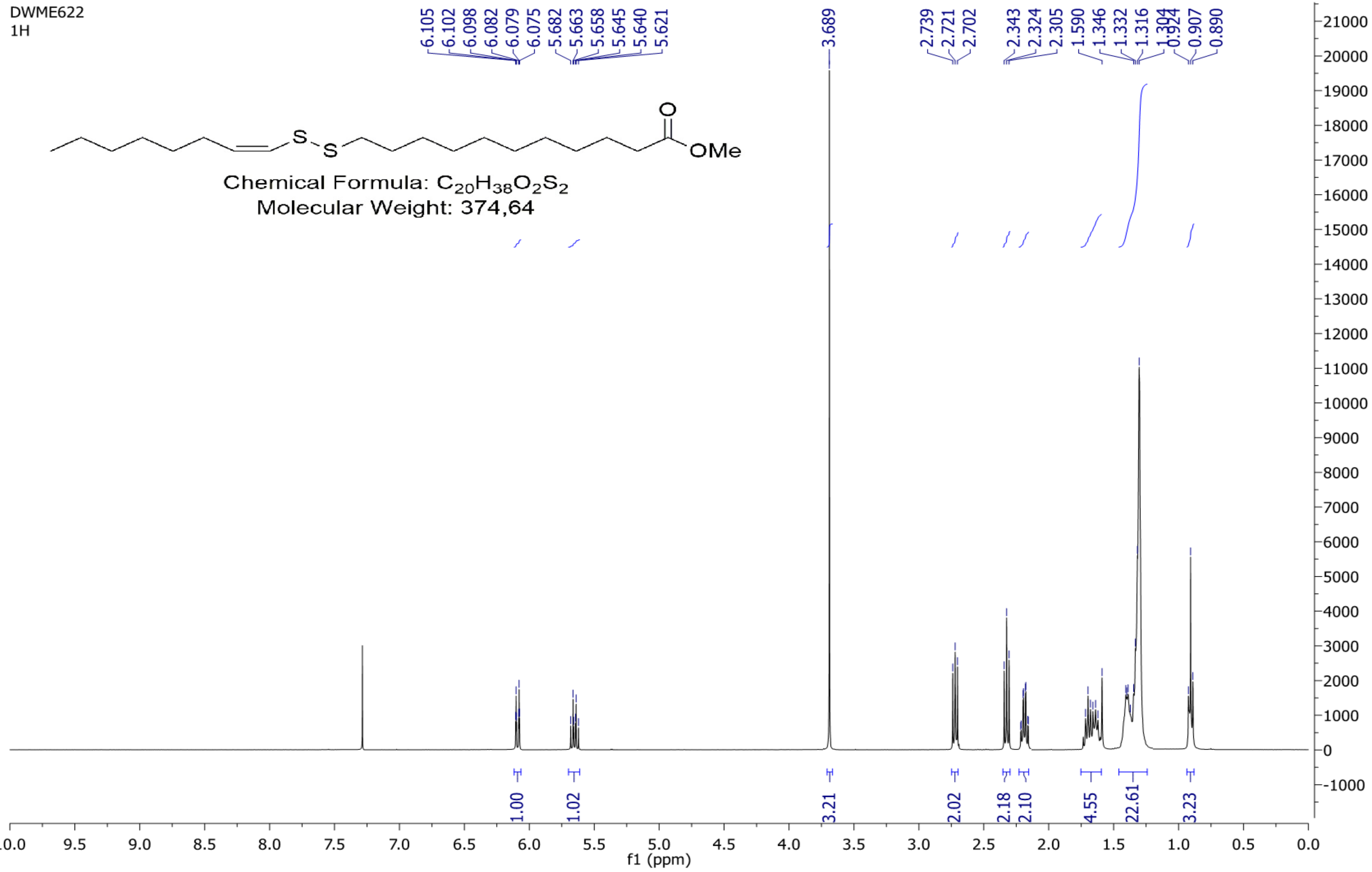
DWME622
1H
6.105
6.102
6.098
6.082
6.079
6.075
5.682
5.663
5.658
5.645
5.640
5.621
3.689
2.739
2.721
2.702
2.343
2.324
2.305
1.590
1.346
1.332
1.316
1.304
0.924
0.907
0.890
S
S
O
OMe
Chemical Formula: $C_{20}H_{38}O_2S_2$
Molecular Weight: 374,64
1.00
1.02
3.21
2.02
2.18
2.10
4.55
22.61
3.23
f1 (ppm)

Z-6c

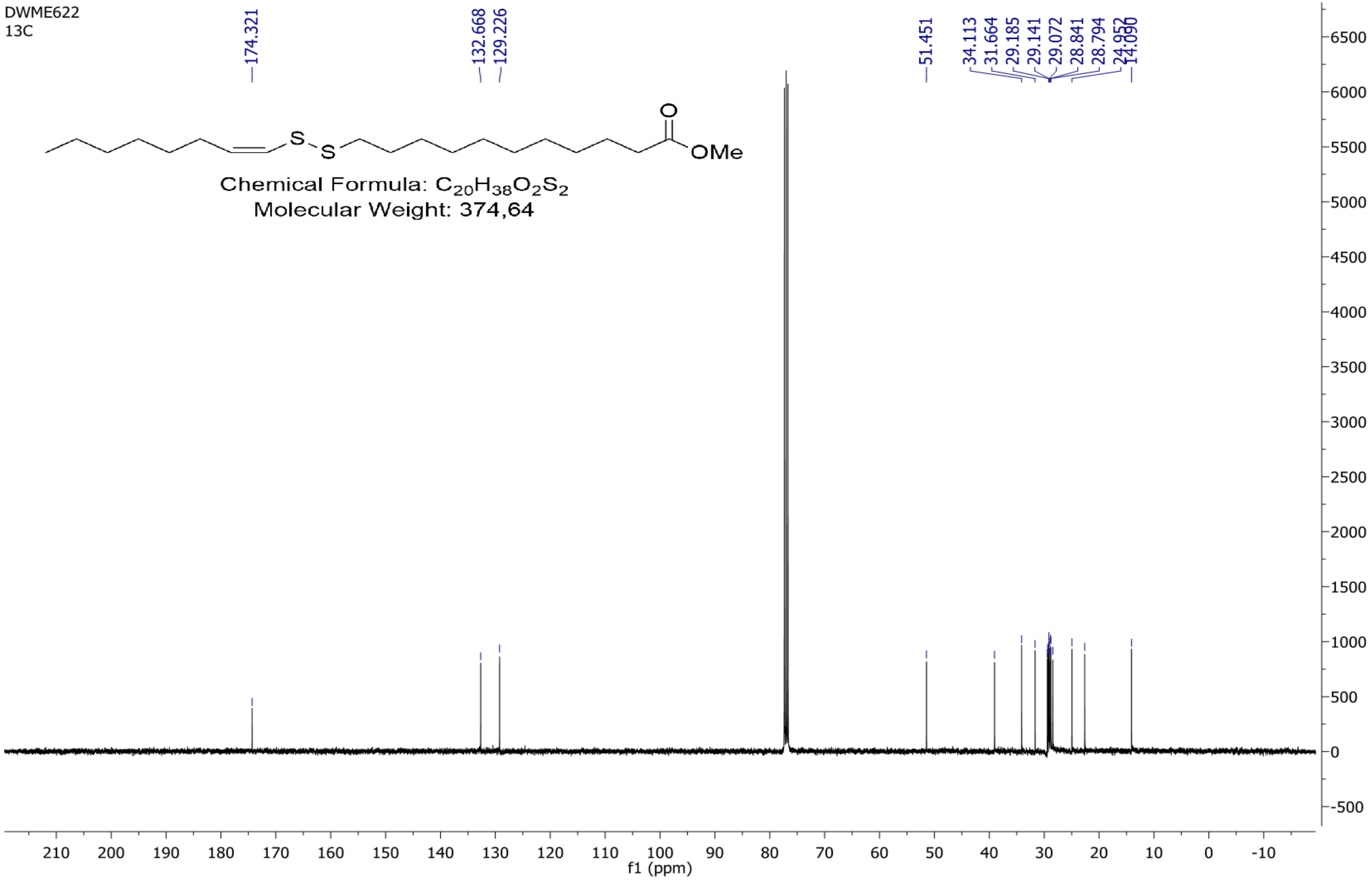

DWME622
13C
174.321
132.668
129.226
51.451
34.113
31.664
29.185
29.141
29.072
28.841
28.794
24.952
14.090
S
S
O
OMe
Chemical Formula: $C_{20}H_{38}O_2S_2$
Molecular Weight: 374,64
f1 (ppm)

IR (ATR) ***Z*-6d**

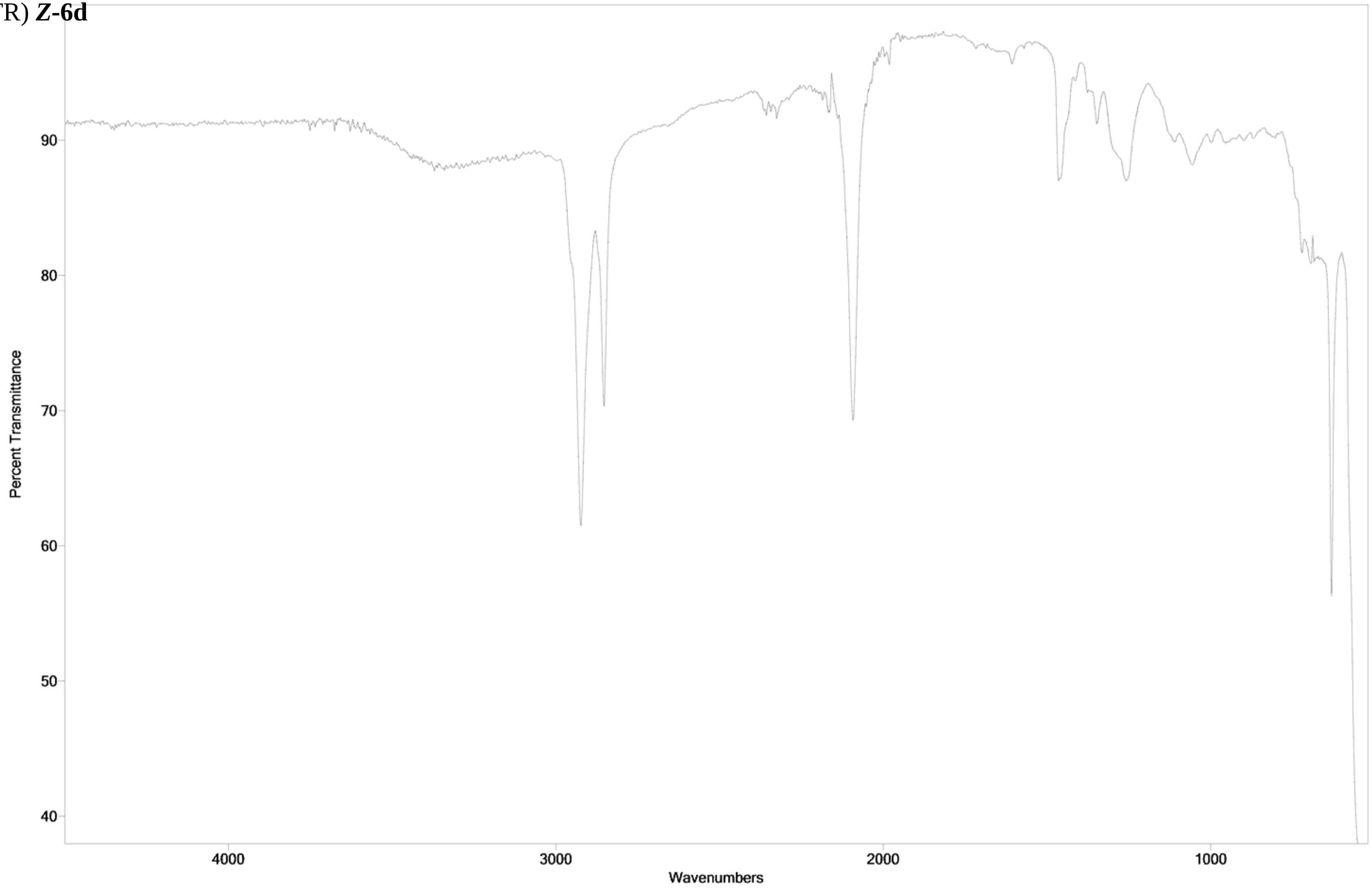

Percent Transmittance
90
80
70
60
50
40
4000
3000
2000
1000
Wavenumbers

Z-6d

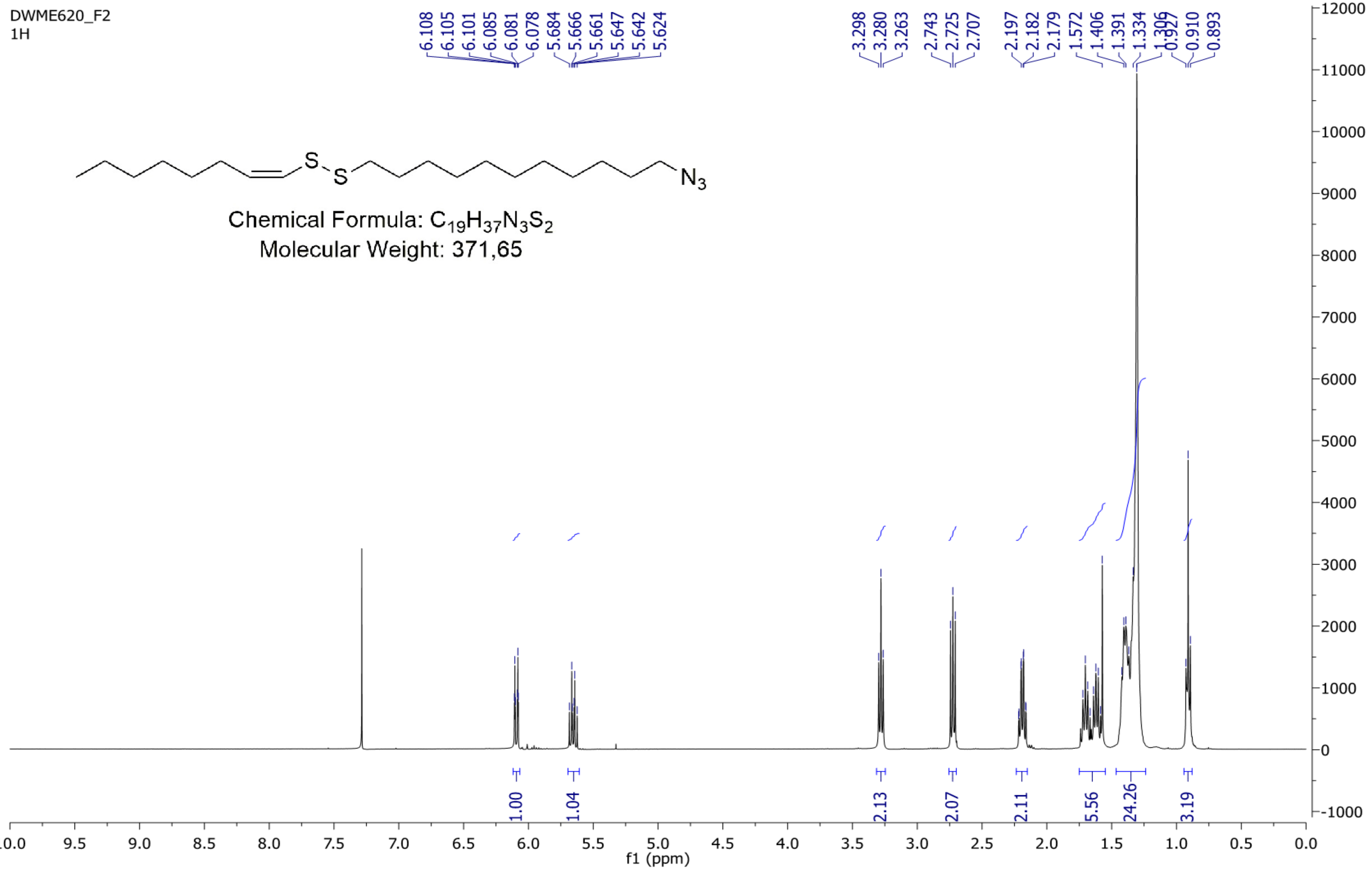

DWME620_F2
1H
6.108
6.105
6.101
6.085
6.081
6.078
5.684
5.666
5.661
5.647
5.642
5.624
3.298
3.280
3.263
2.743
2.725
2.707
2.197
2.182
2.179
1.572
1.406
1.391
1.334
1.306
0.927
0.910
0.893
N3
Chemical Formula: C19H37N3S2
Molecular Weight: 371,65
1.00
1.04
2.13
2.07
2.11
5.56
24.26
3.19
f1 (ppm)

Z-6d

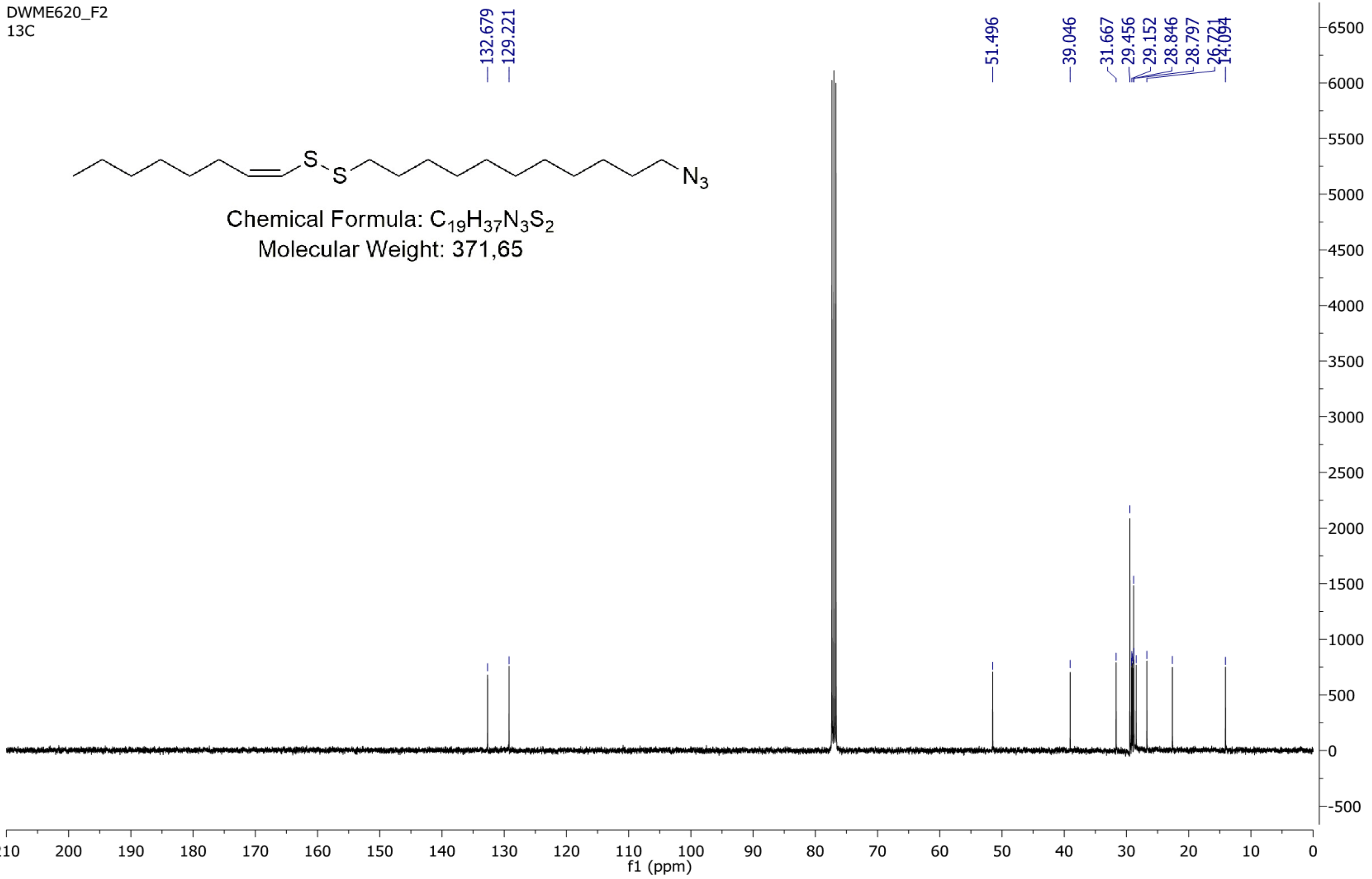
DWME620_F2
13C
132.679
129.221
51.496
39.046
31.667
29.456
29.152
28.846
28.797
26.721
14.094
S
S
N3
Chemical Formula: C19H37N3S2
Molecular Weight: 371,65
6500
6000
5500
5000
4500
4000
3500
3000
2500
2000
1500
1000
500
0
-500
210 200 190 180 170 160 150 140 130 120 110 100 90 80 70 60 50 40 30 20 10 0
f1 (ppm)

IR (ATR) ***Z*-6e**

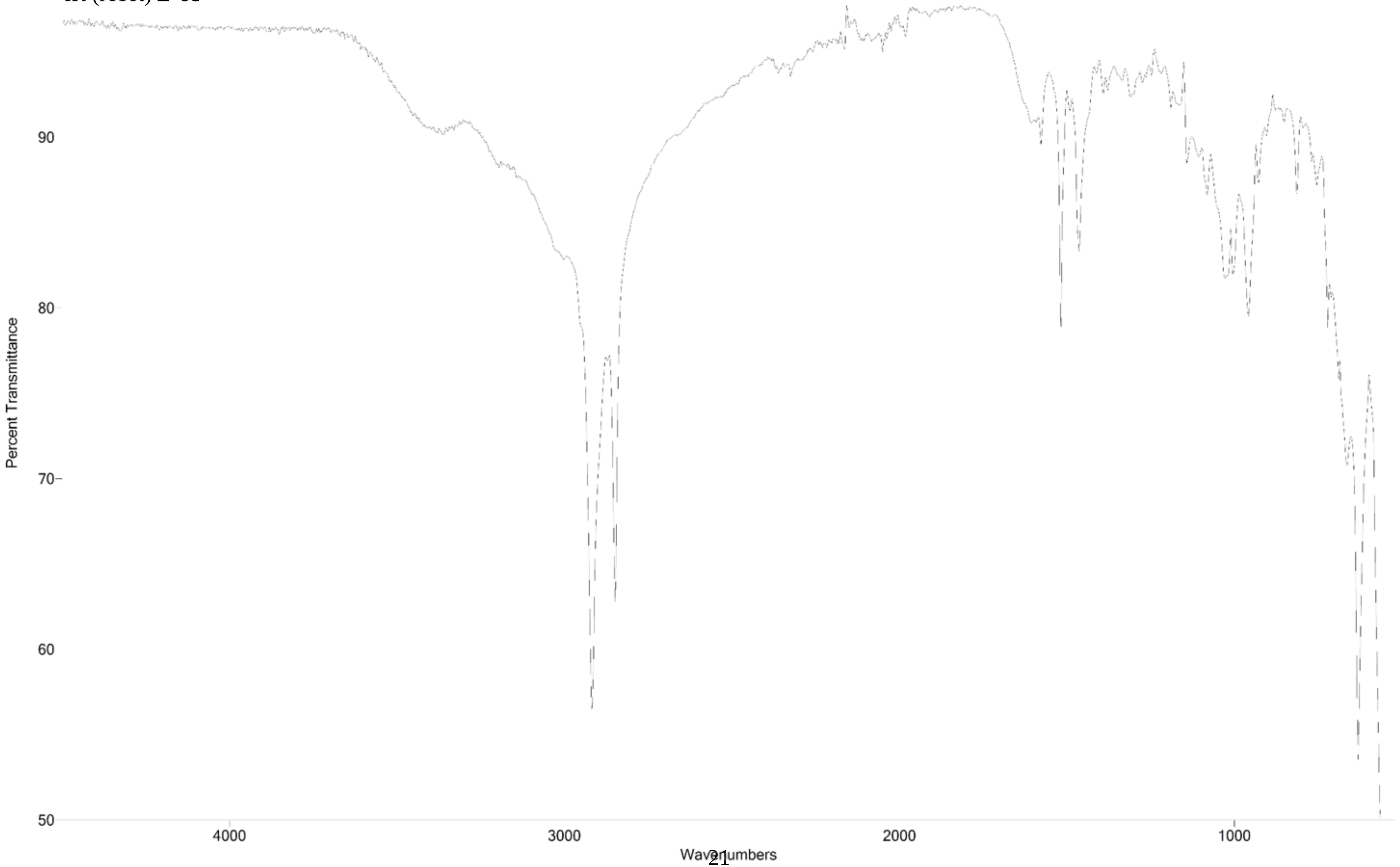
Percent Transmittance
90
80
70
60
50
4000
3000
2000
1000
Wavenumbers

***Z*-6e**

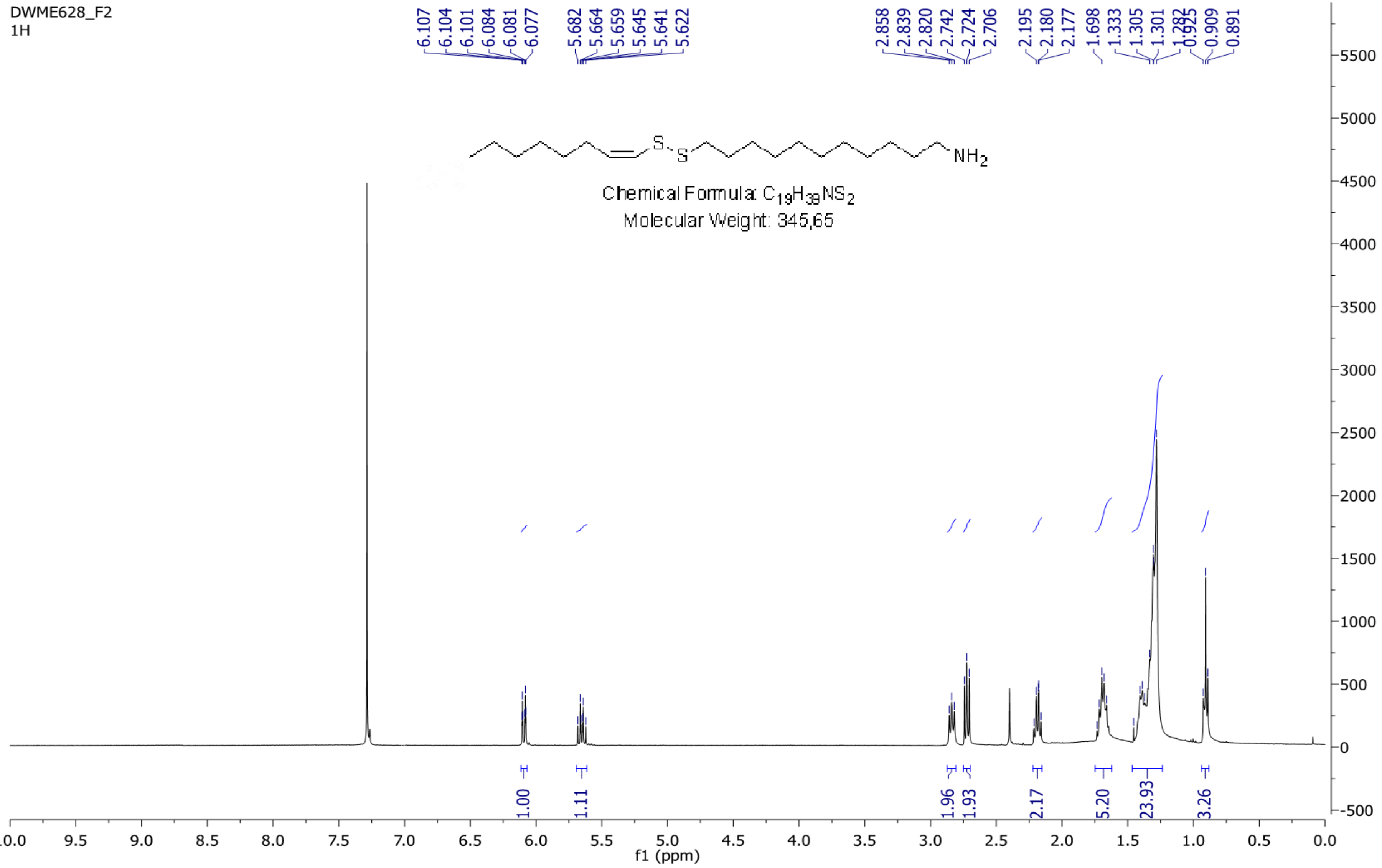
DWME628_F2
1H
Chemical Formula: C19H39NS2
Molecular Weight: 345,65
NH2
S
S
6.107
6.104
6.101
6.084
6.081
6.077
5.682
5.664
5.659
5.645
5.641
5.622
2.858
2.839
2.820
2.742
2.724
2.706
2.195
2.180
2.177
1.698
1.333
1.305
1.301
1.282
0.925
0.909
0.891
1.00
1.11
1.96
1.93
2.17
5.20
23.93
3.26
f1 (ppm)

***Z*-6e**

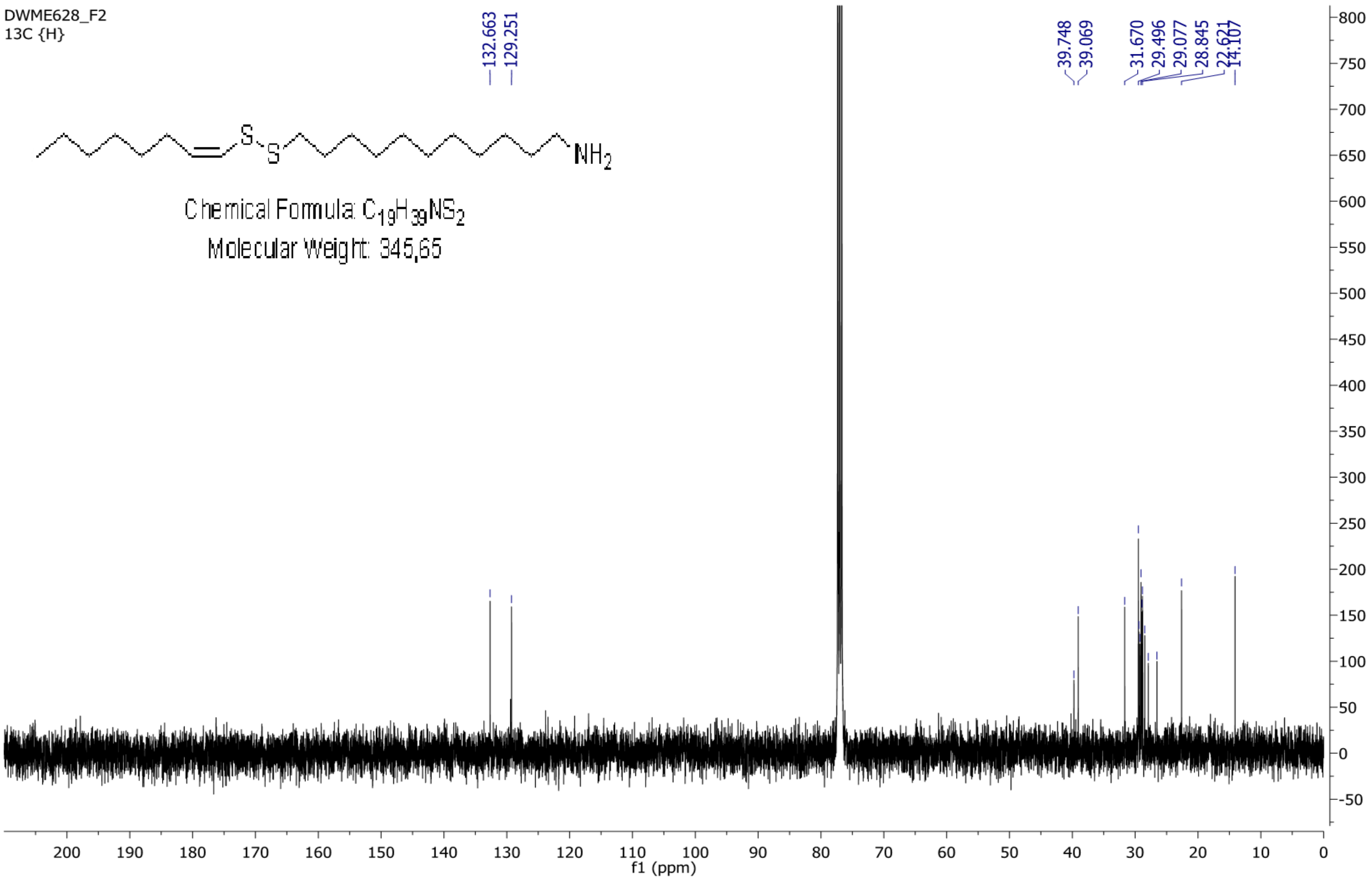

DWME628_F2
13C {H}
132.663
129.251
39.748
39.069
31.670
29.496
29.077
28.845
22.621
14.107
Chemical Formula: C19H39NS2
Molecular Weight: 345,65
S
S
NH2
f1 (ppm)

Z-6f

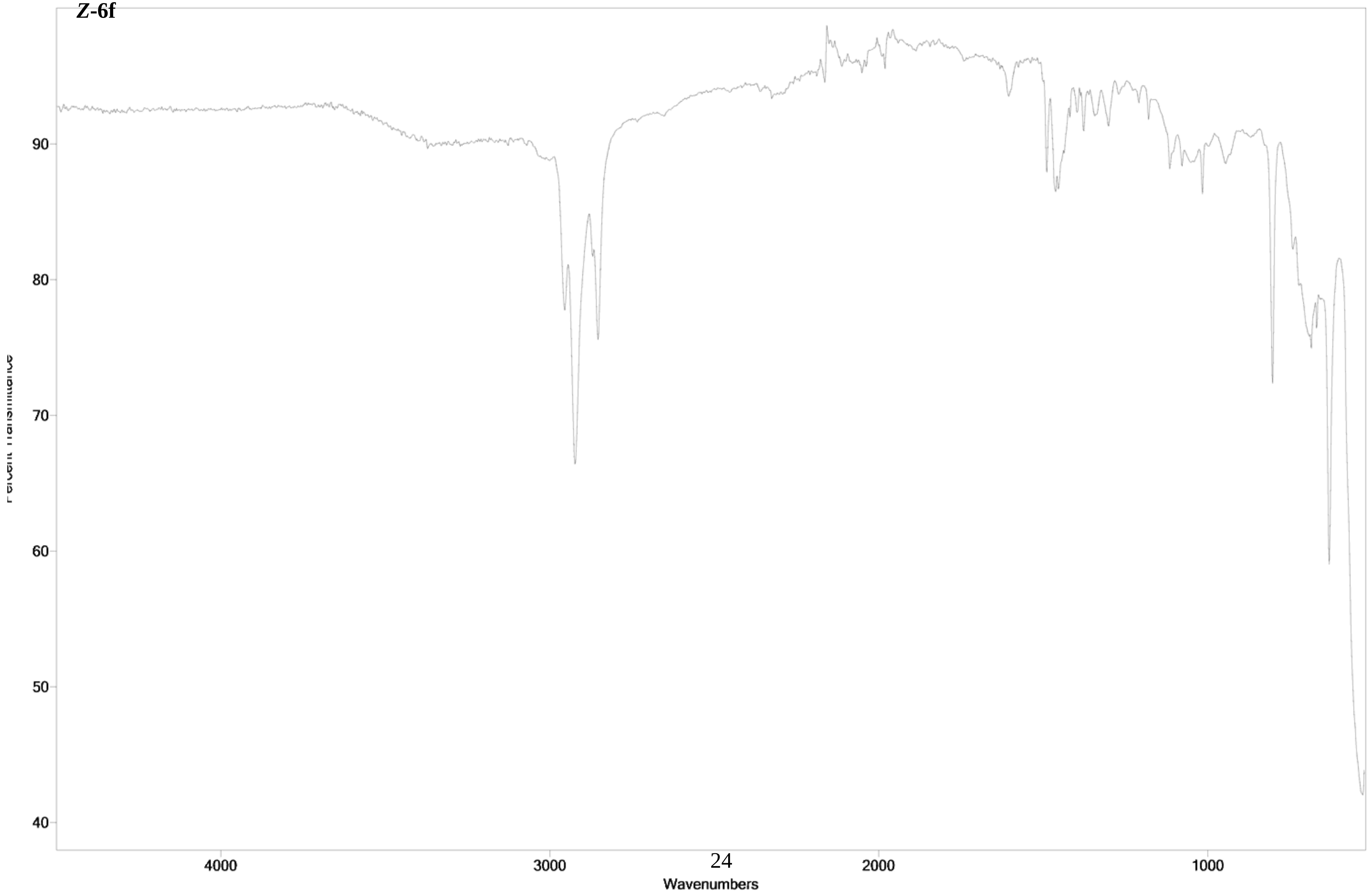

Z-6f

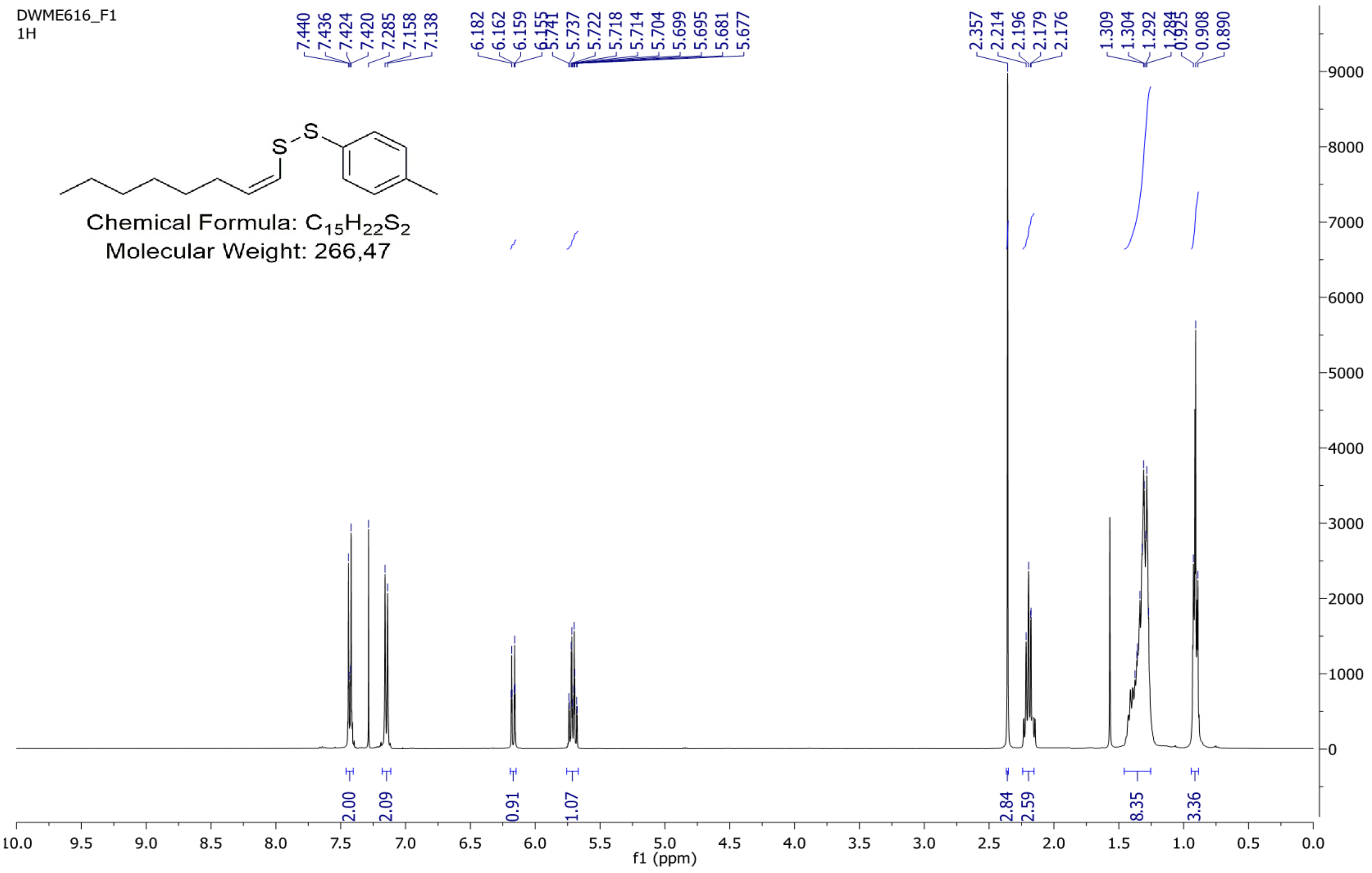

DWME616_F1
1H
Chemical Formula: $C_{15}H_{22}S_2$
Molecular Weight: 266,47
f1 (ppm)

Z-6f

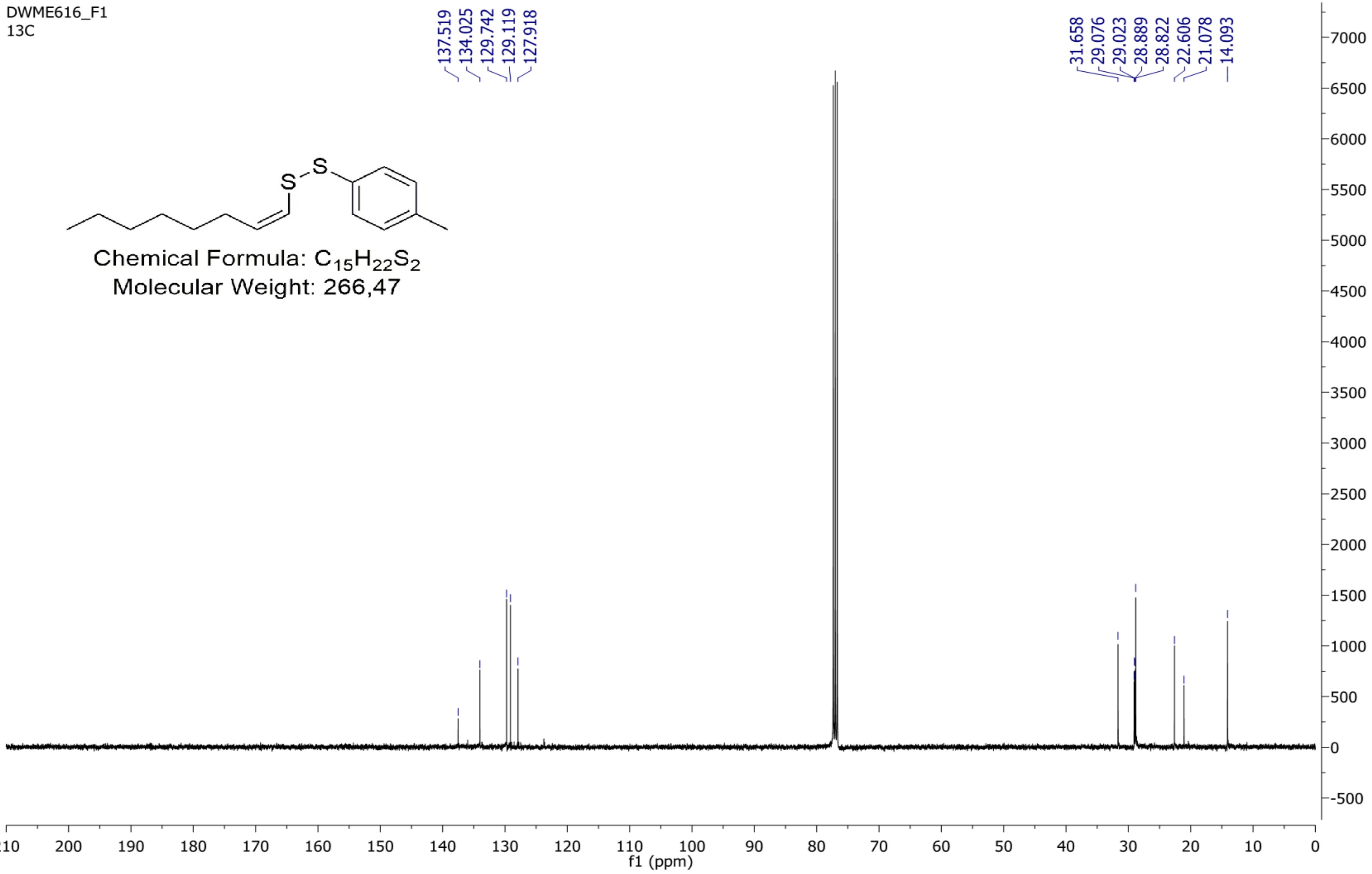
DWME616_F1
13C
137.519
134.025
129.742
129.119
127.918
31.658
29.076
29.023
28.889
28.822
22.606
21.078
14.093
Chemical Formula: C15H22S2
Molecular Weight: 266,47
f1 (ppm)

IR (ATR) **Z-6g**

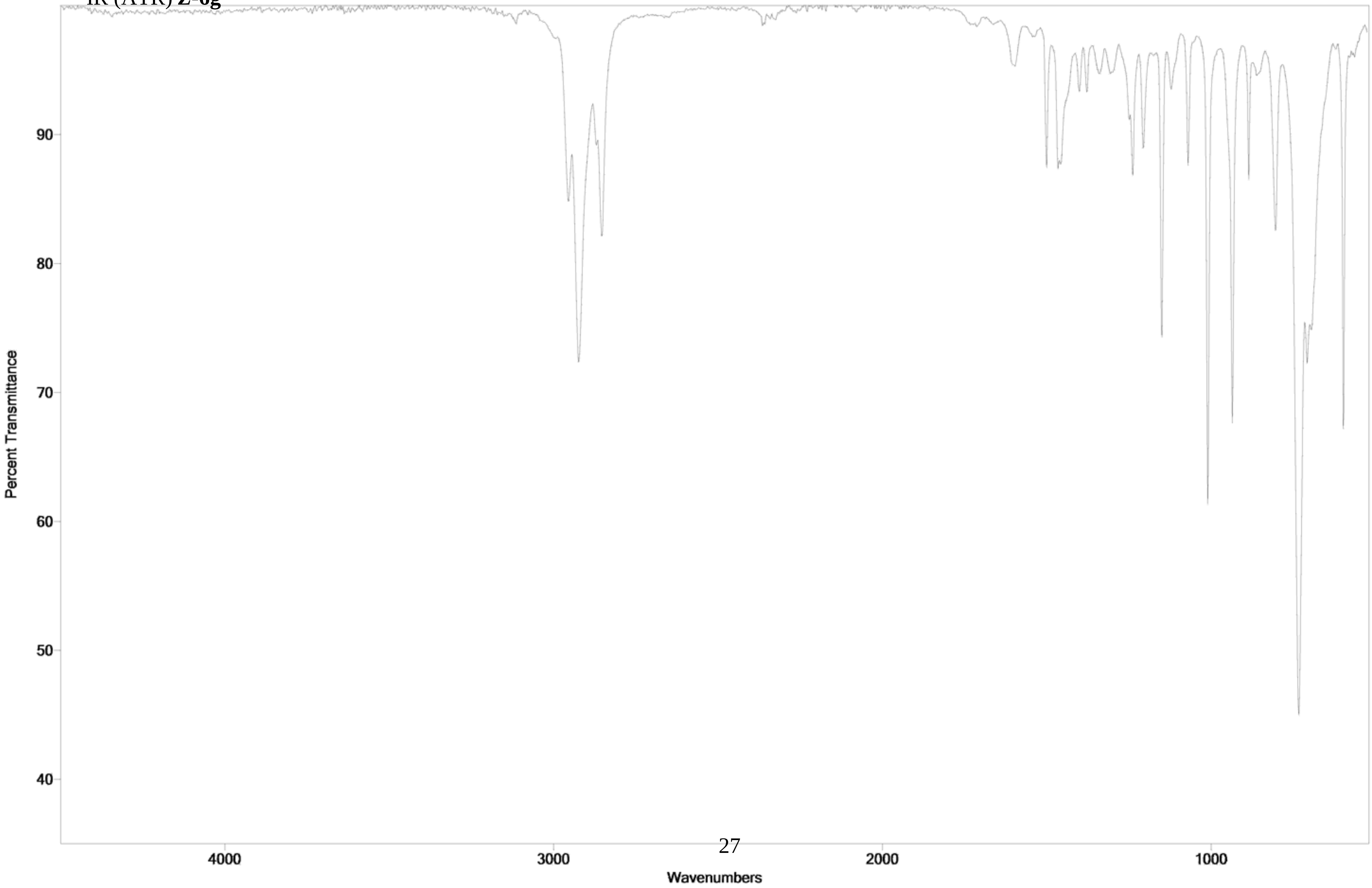
Percent Transmittance
90
80
70
60
50
40
4000
3000
2000
1000
Wavenumbers

Z-6g

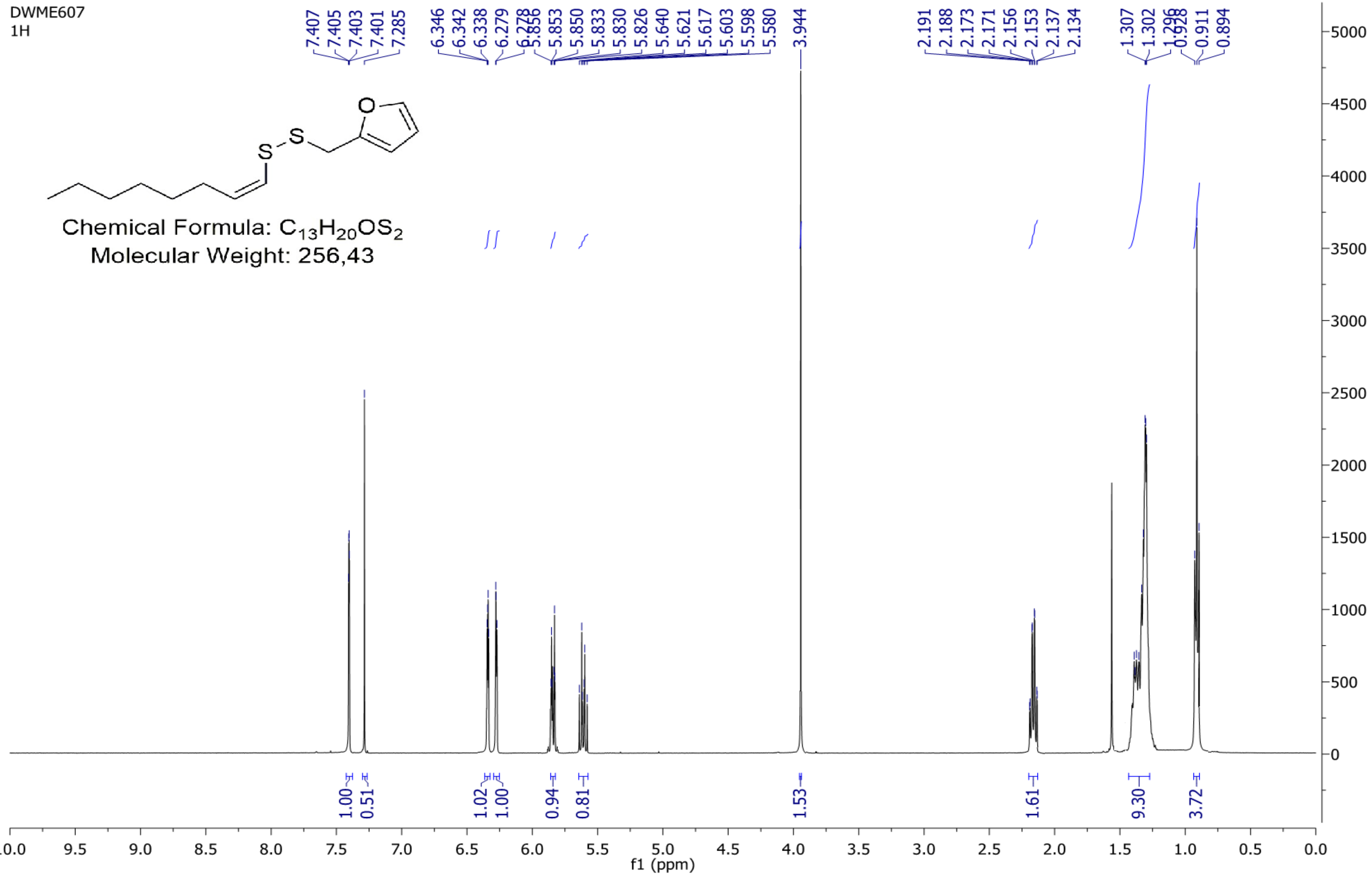
DWME607
1H
Chemical Formula: C13H20OS2
Molecular Weight: 256,43
7.407 7.405 7.403 7.401 7.285
6.346 6.342 6.338 6.279
5.853 5.850 5.833 5.830 5.826 5.640 5.621 5.617 5.603 5.598 5.580
3.944
2.191 2.188 2.173 2.171 2.156 2.153 2.137 2.134
1.307 1.302 0.911 0.894
1.00 0.51 1.02 1.00 0.94 0.81 1.53 1.61 9.30 3.72
f1 (ppm)

Z-6g

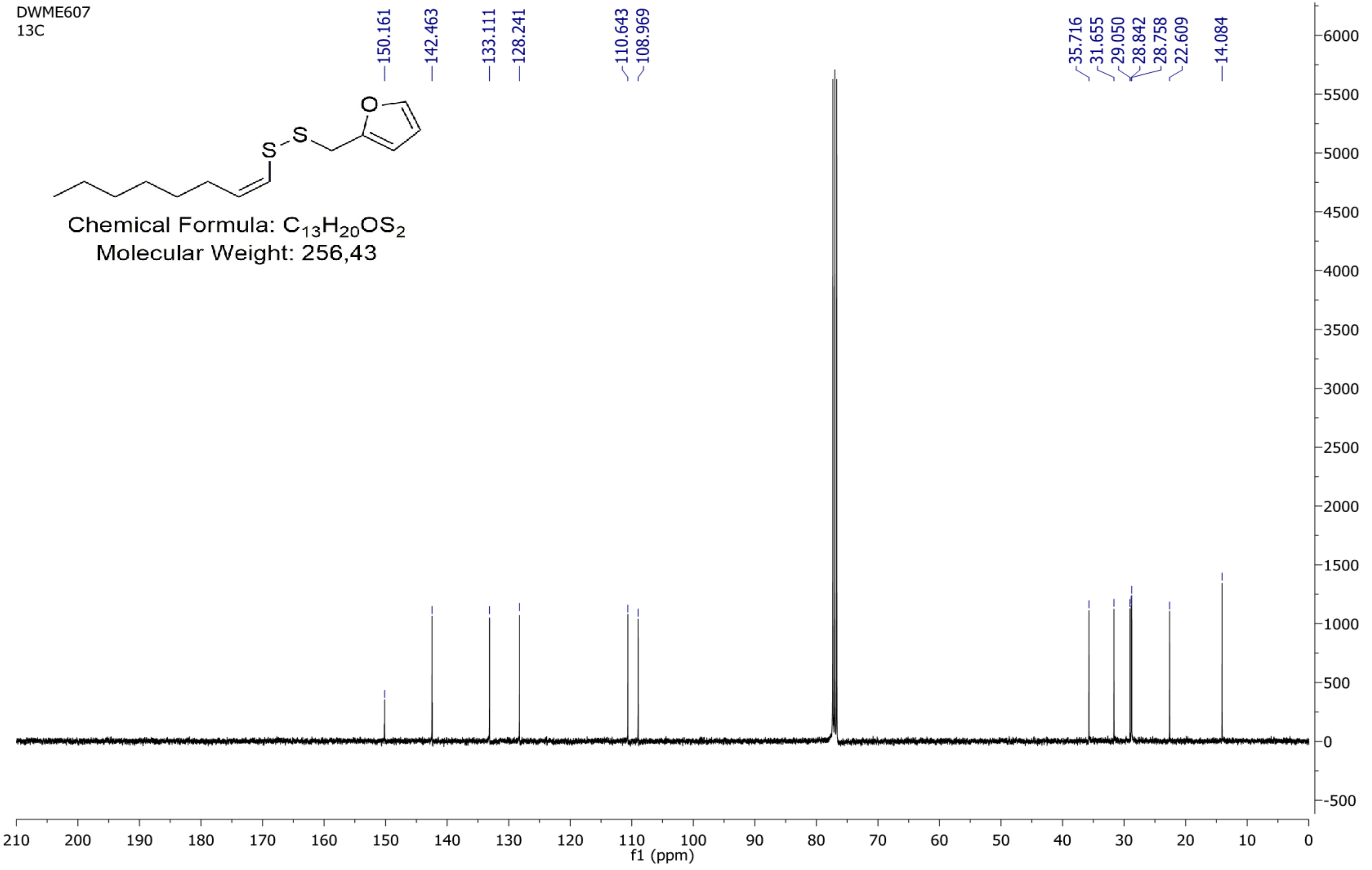
DWME607
13C
150.161
142.463
133.111
128.241
110.643
108.969
35.716
31.655
29.050
28.842
28.758
22.609
14.084
Chemical Formula: C13H20OS2
Molecular Weight: 256,43
f1 (ppm)

IR (ATR) ***Z*-6h**

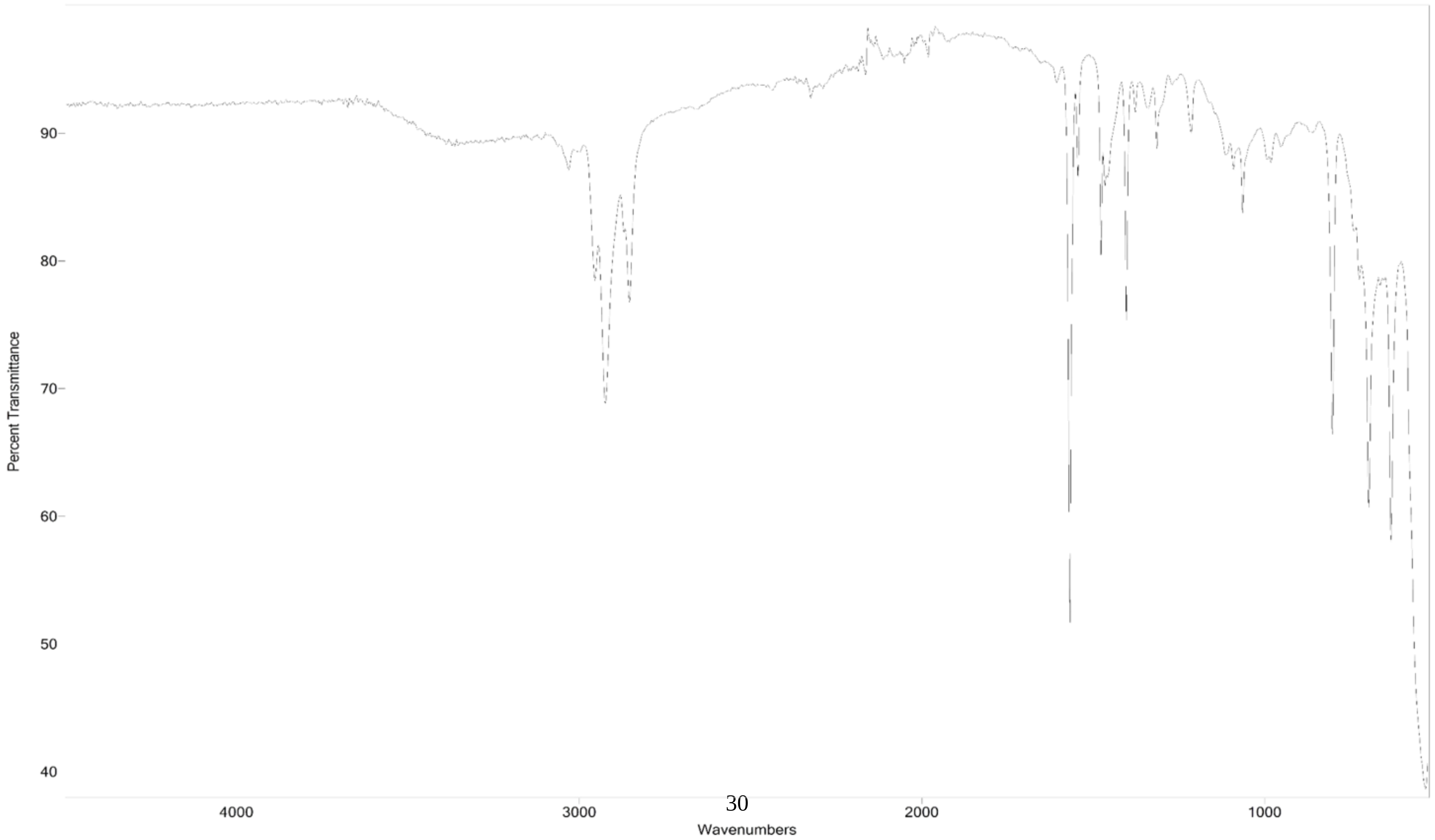

Percent Transmittance
90
80
70
60
50
40
4000
3000
2000
1000
Wavenumbers

Z-6h

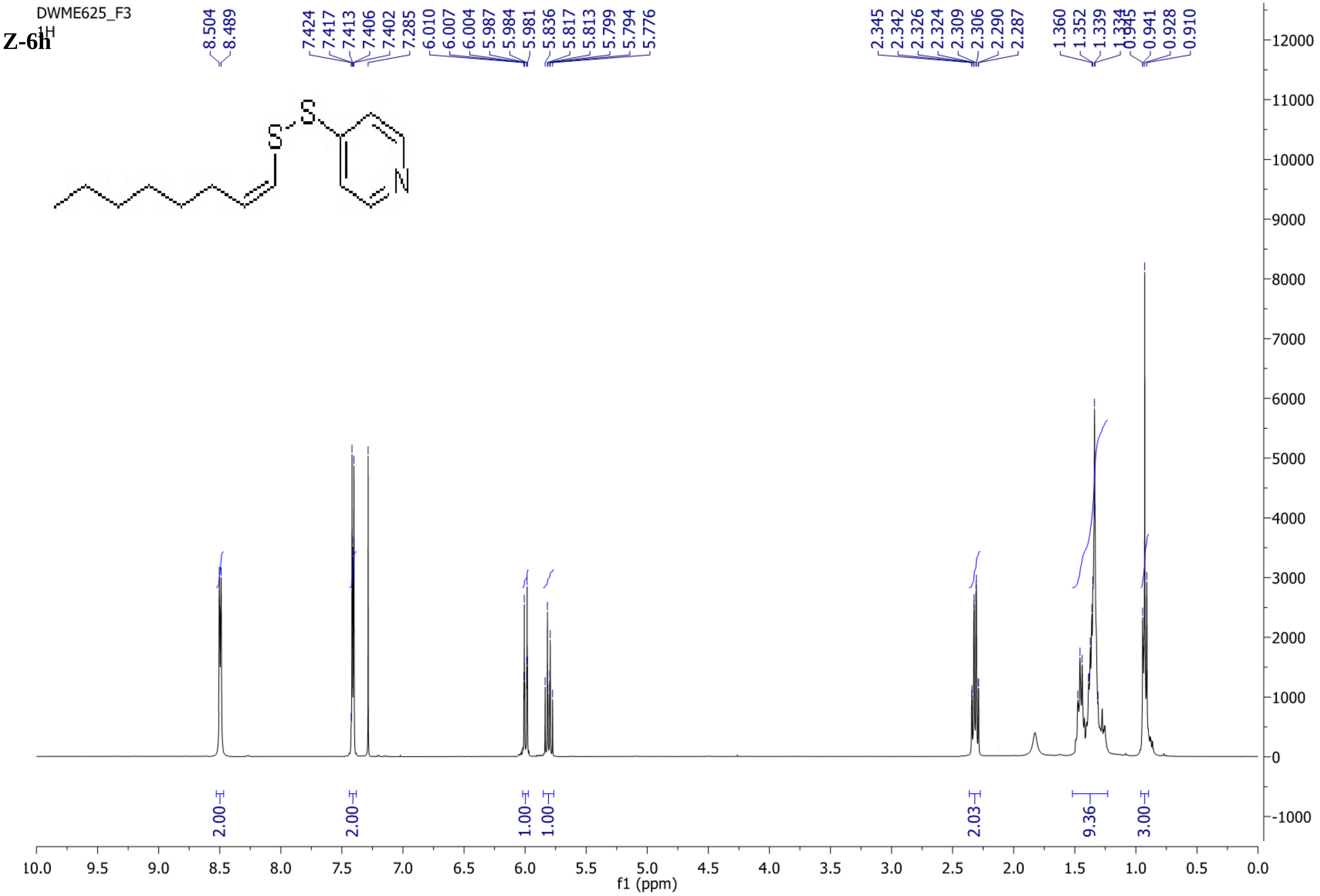
DWME625_F3
1H
8.504
8.489
7.424
7.417
7.413
7.406
7.402
7.285
6.010
6.007
6.004
5.987
5.984
5.981
5.836
5.817
5.813
5.799
5.794
5.776
2.345
2.342
2.326
2.324
2.309
2.306
2.290
2.287
1.360
1.352
1.339
1.334
0.945
0.941
0.928
0.910
S
S
N
2.00
2.00
1.00
1.00
2.03
9.36
3.00
f1 (ppm)

***Z*-6h**

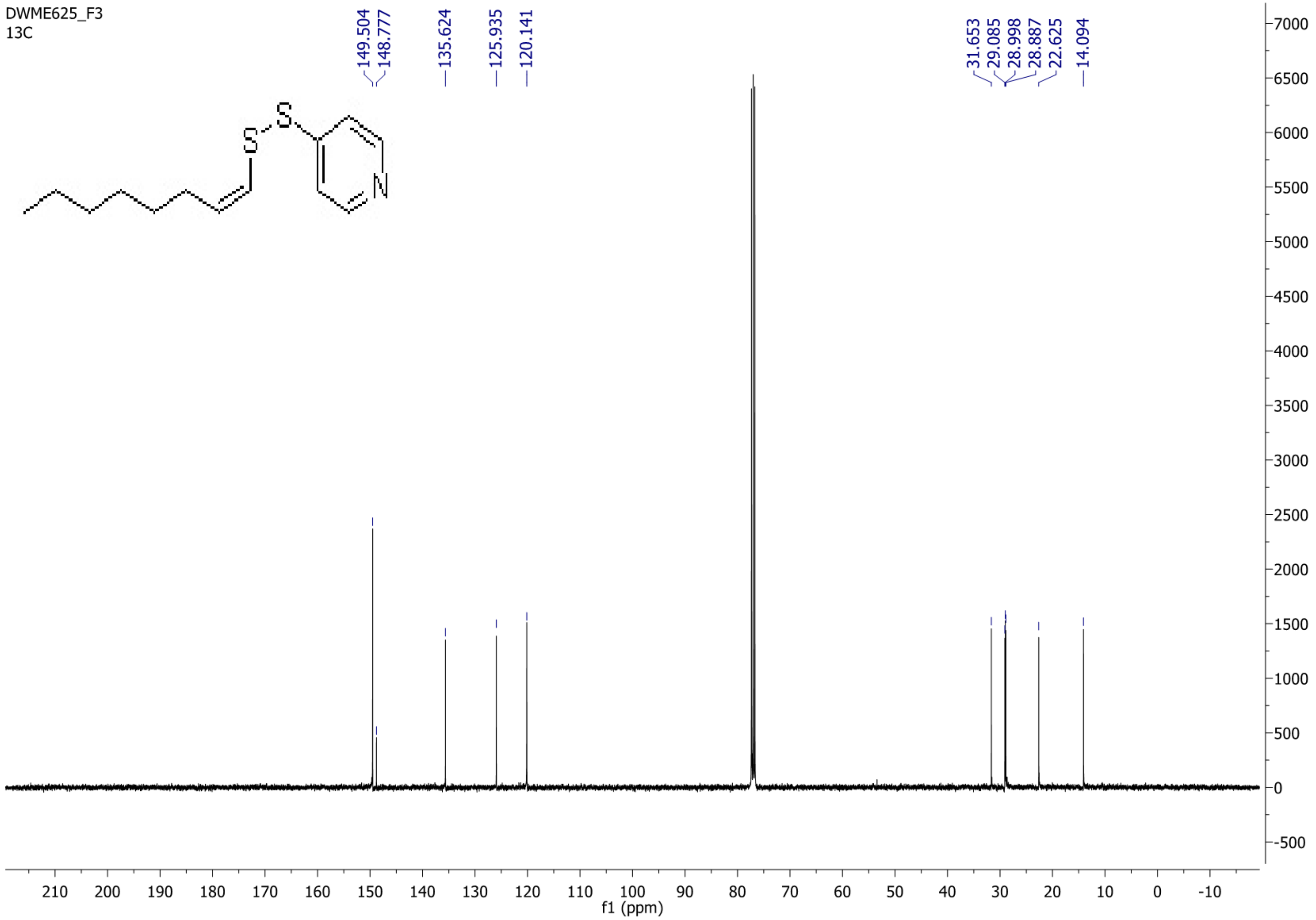
DWME625_F3
13C
149.504
148.777
135.624
125.935
120.141
31.653
29.085
28.998
28.887
22.625
14.094
S
S
N
7000
6500
6000
5500
5000
4500
4000
3500
3000
2500
2000
1500
1000
500
0
-500
210 200 190 180 170 160 150 140 130 120 110 100 90 80 70 60 50 40 30 20 10 0 -10
f1 (ppm)

Z-6i

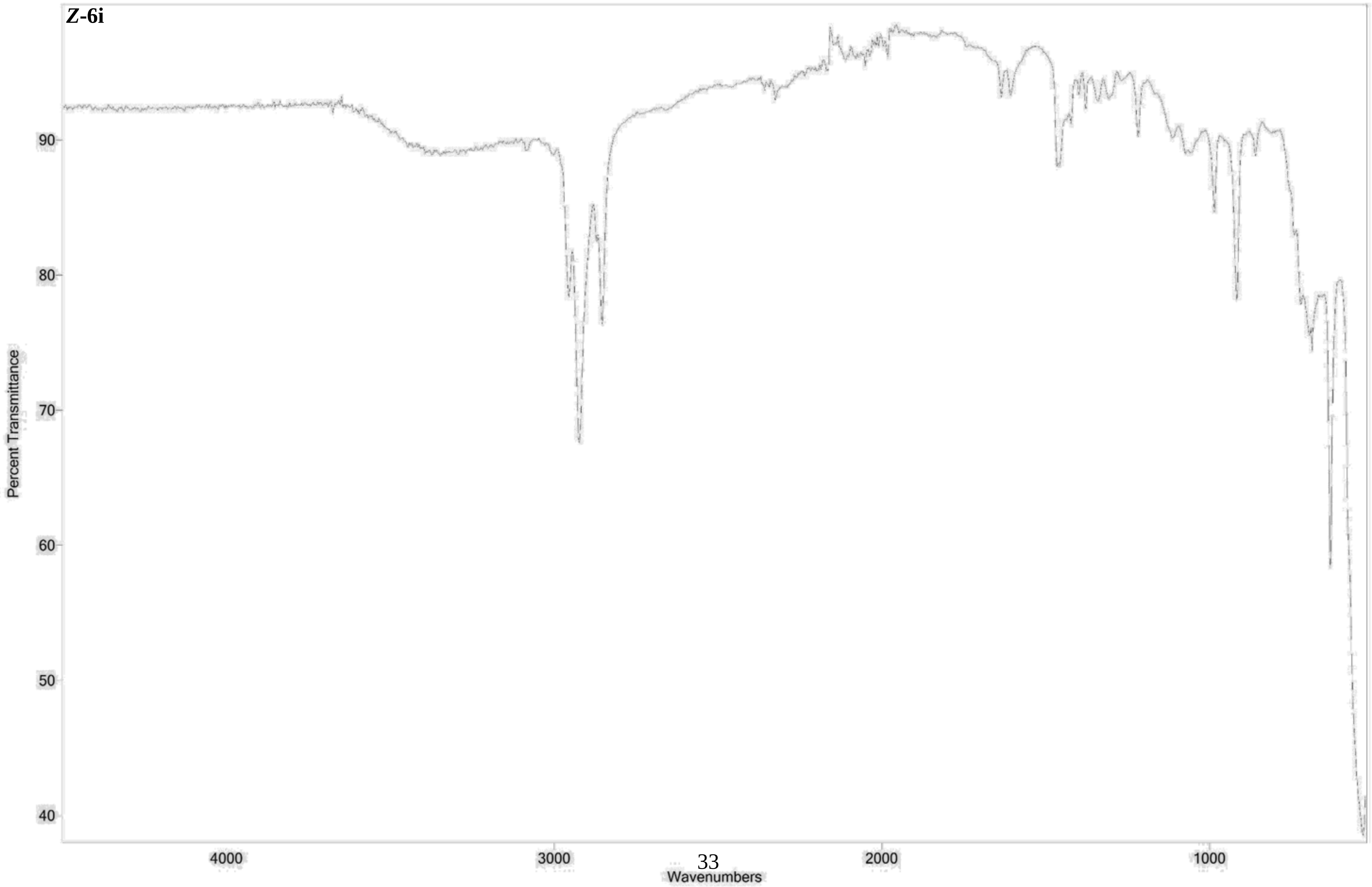

Percent Transmittance
90
80
70
60
50
40
4000
3000
2000
1000
Wavenumbers

Z-6i

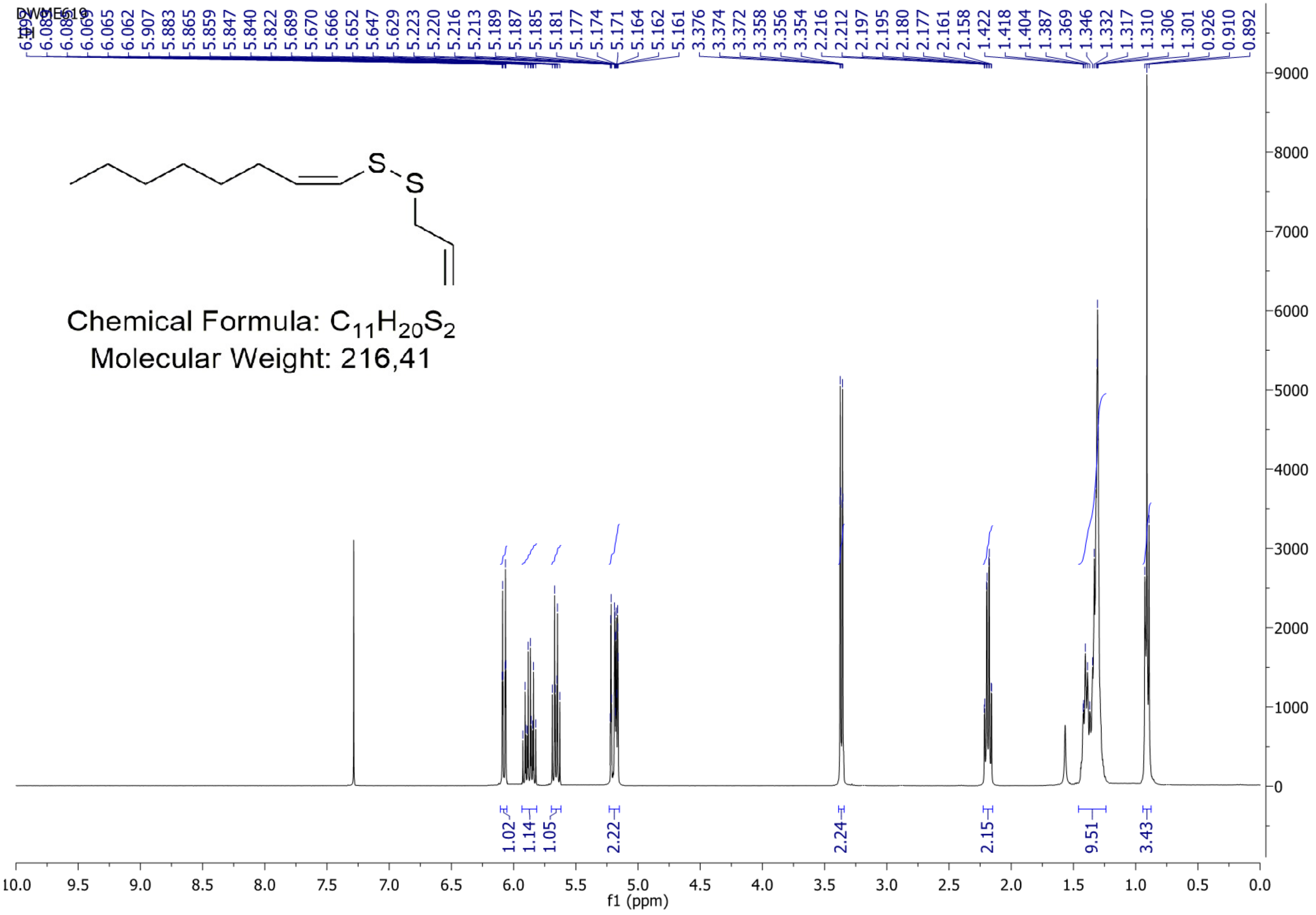

Z-6i

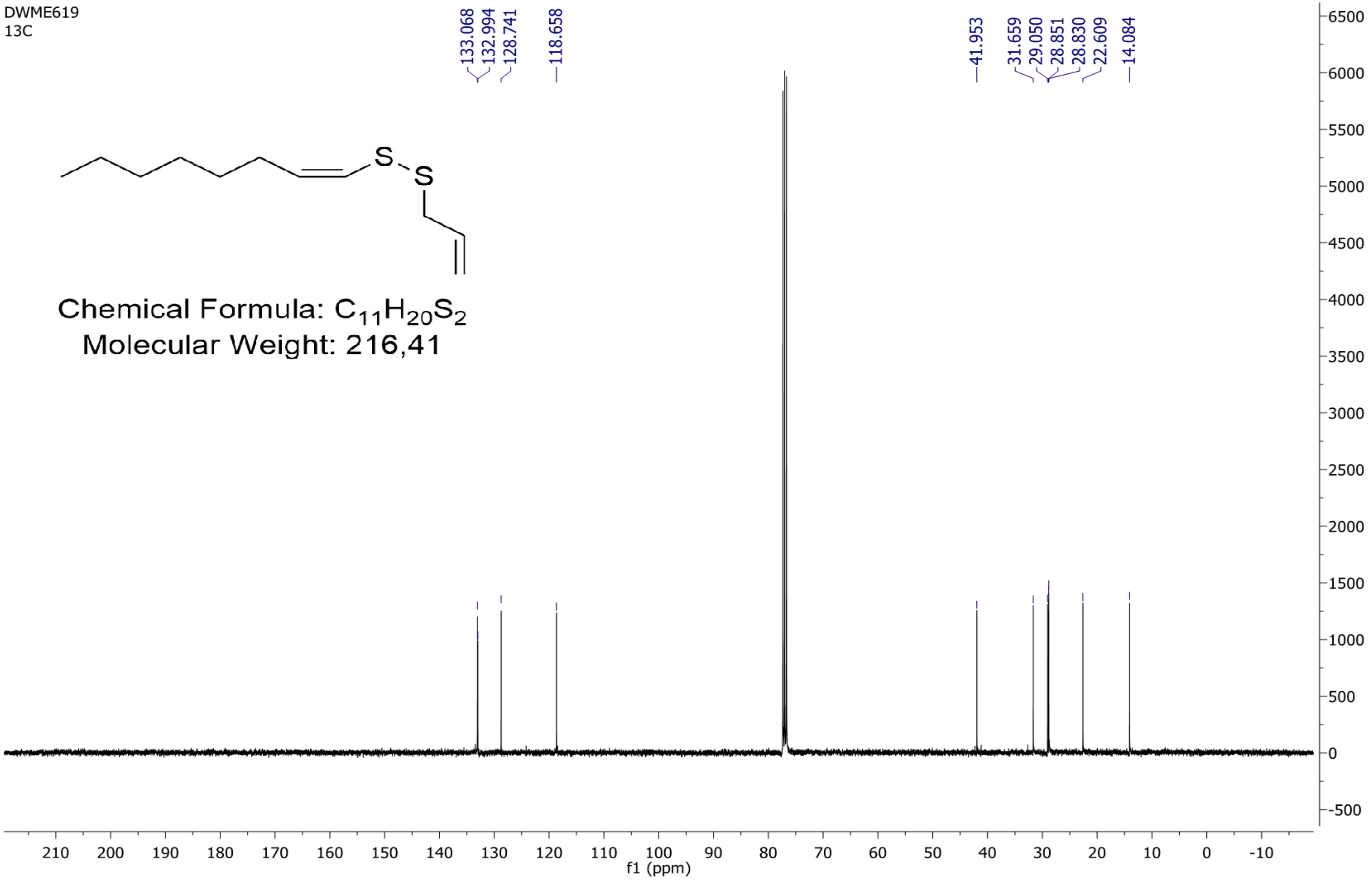
DWME619
13C
133.068
132.994
128.741
118.658
41.953
31.659
29.050
28.851
28.830
22.609
14.084
Chemical Formula: $C_{11}H_{20}S_2$
Molecular Weight: 216,41
f1 (ppm)

Z-6j

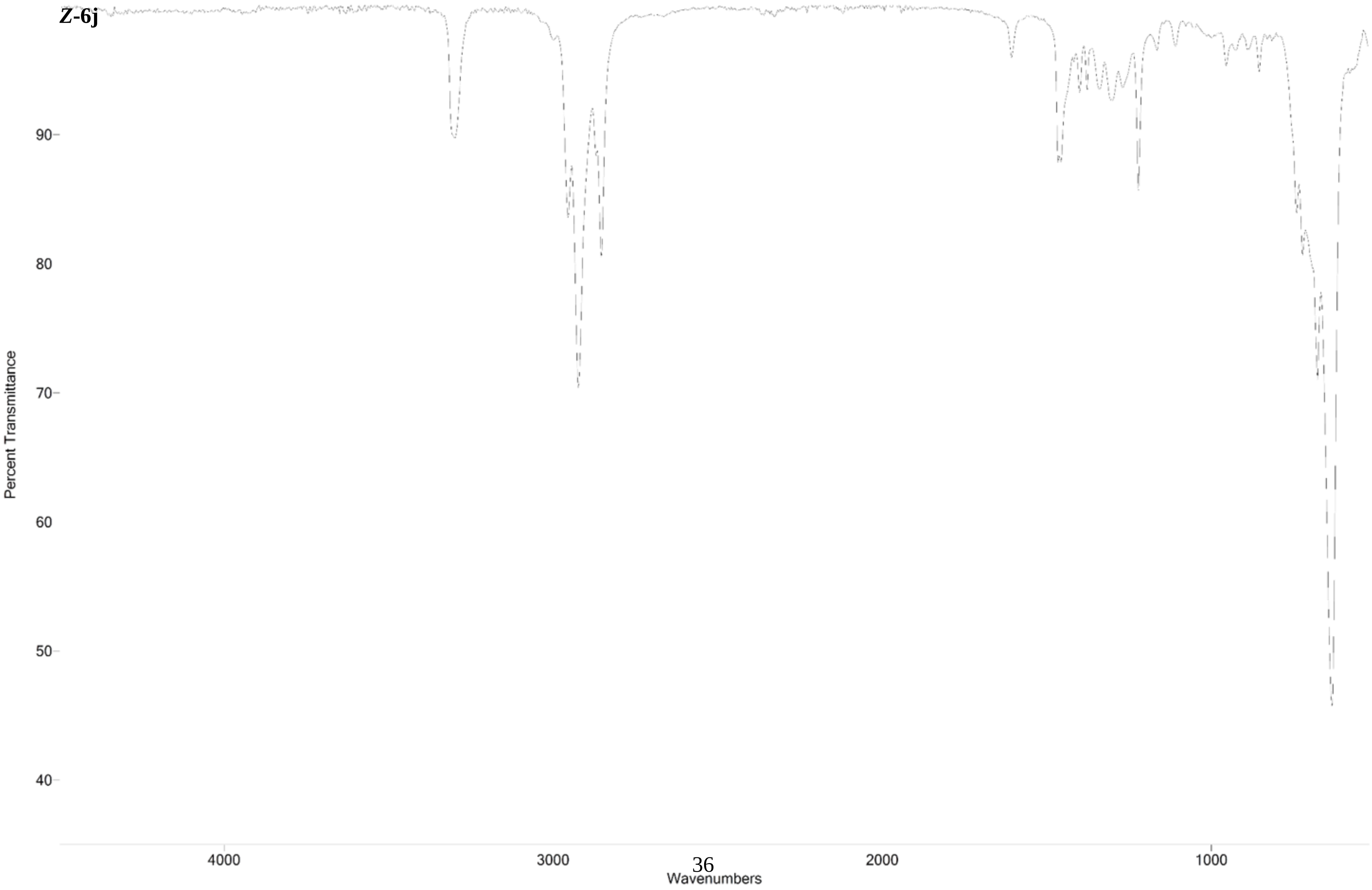

Percent Transmittance
90
80
70
60
50
40
4000
3000
2000
1000
Wavenumbers

Z-6j

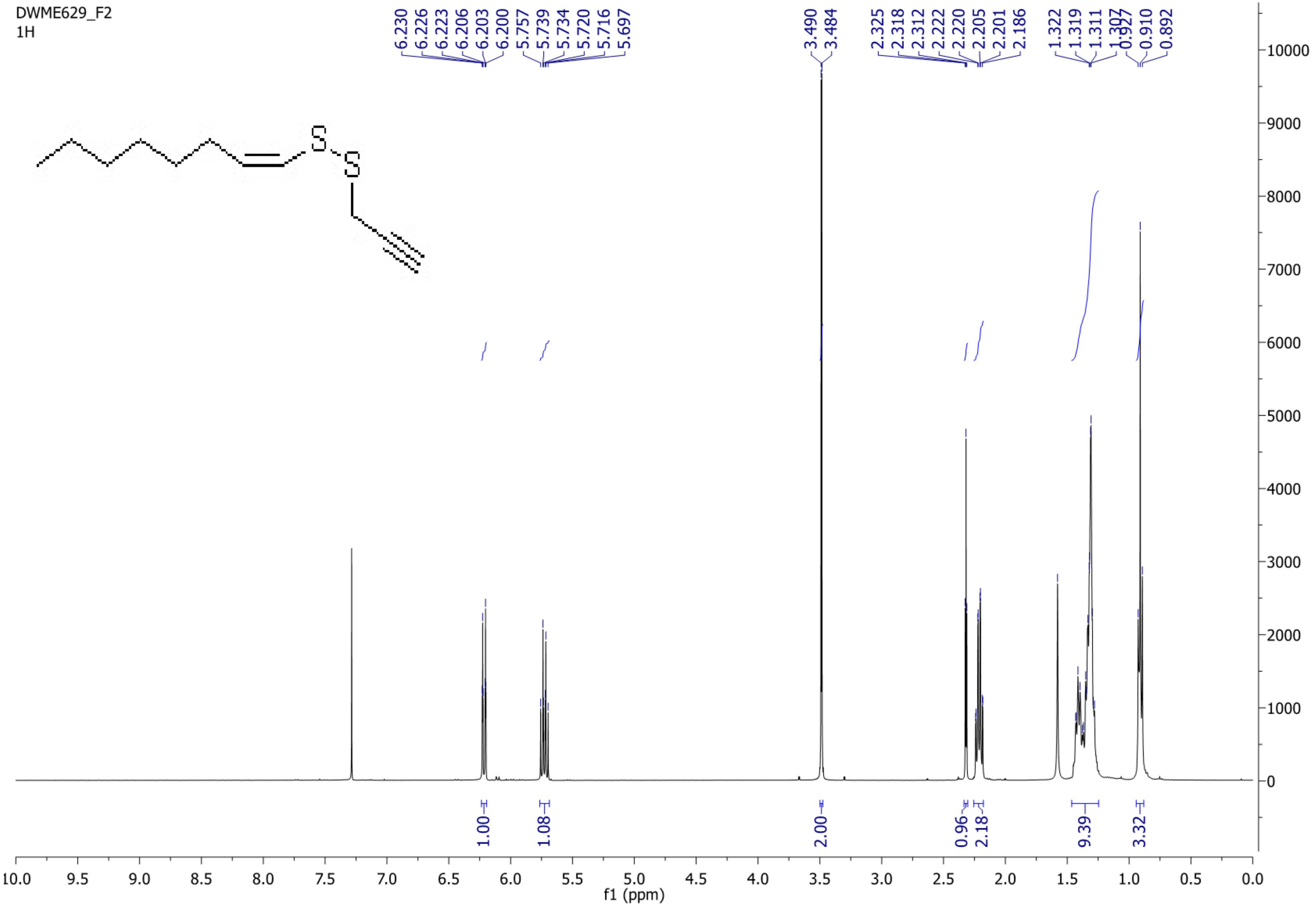

DWME629_F2
1H
6.230
6.226
6.223
6.206
6.203
6.200
5.757
5.739
5.734
5.720
5.716
5.697
3.490
3.484
2.325
2.318
2.312
2.222
2.220
2.205
2.201
2.186
1.322
1.319
1.311
1.307
0.927
0.910
0.892
1.00
1.08
2.00
0.96
2.18
9.39
3.32
f1 (ppm)

Z-6j

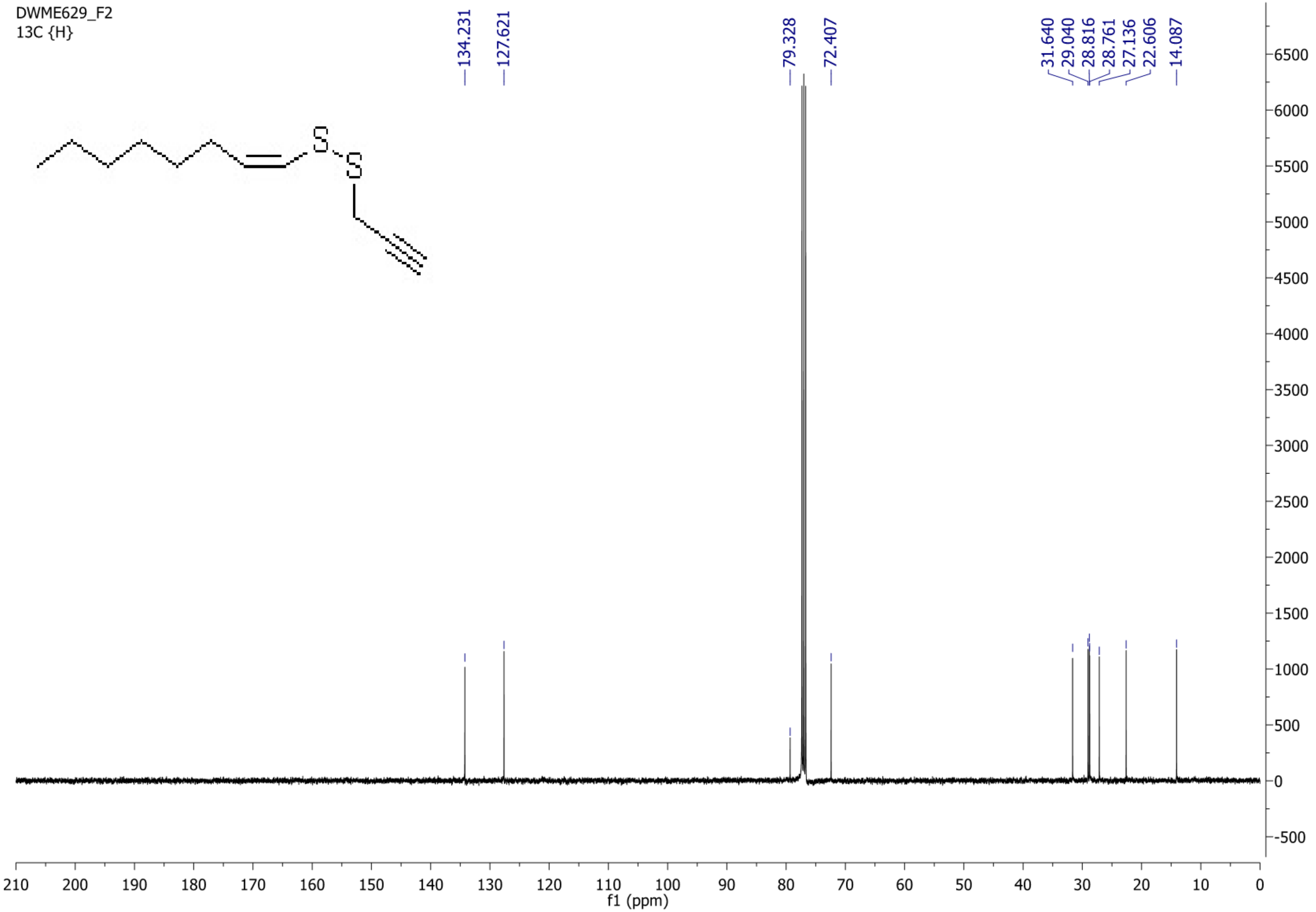
DWME629_F2
13C {H}
134.231
127.621
79.328
72.407
31.640
29.040
28.816
28.761
27.136
22.606
14.087
S
S
6500
6000
5500
5000
4500
4000
3500
3000
2500
2000
1500
1000
500
0
-500
210
200
190
180
170
160
150
140
130
120
110
100
90
80
70
60
50
40
30
20
10
0
f1 (ppm)

IR (ATR) **Z-6k**

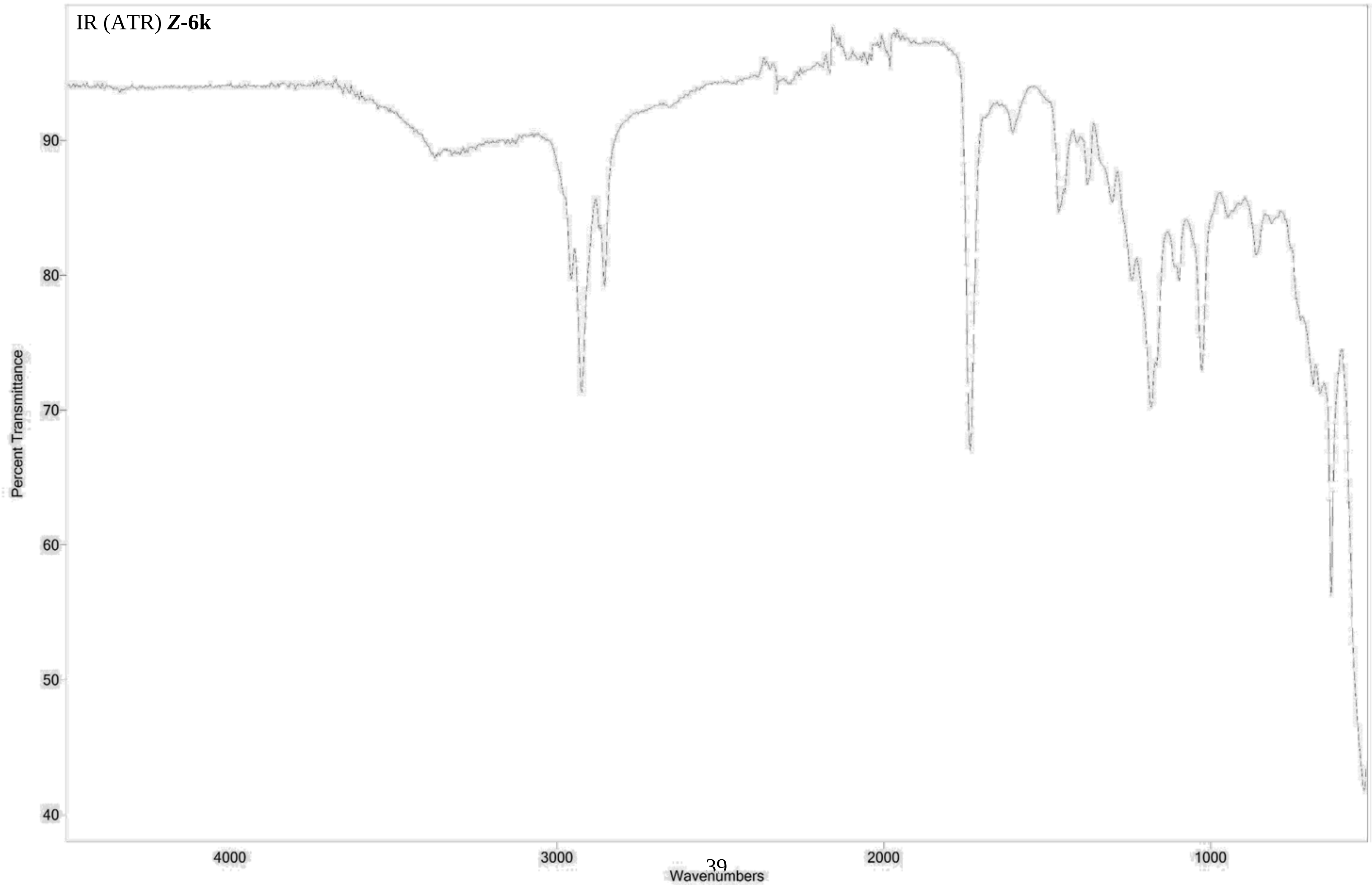

Percent Transmittance
90
80
70
60
50
40
4000
3000
2000
1000
Wavenumbers

Z-6k

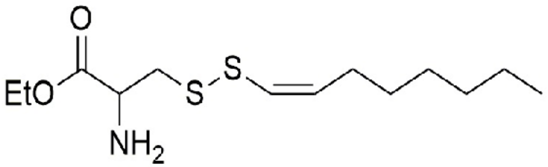

Chemical Formula: $C_{13}H_{25}NO_2S_2$

Molecular Weight: 291,47

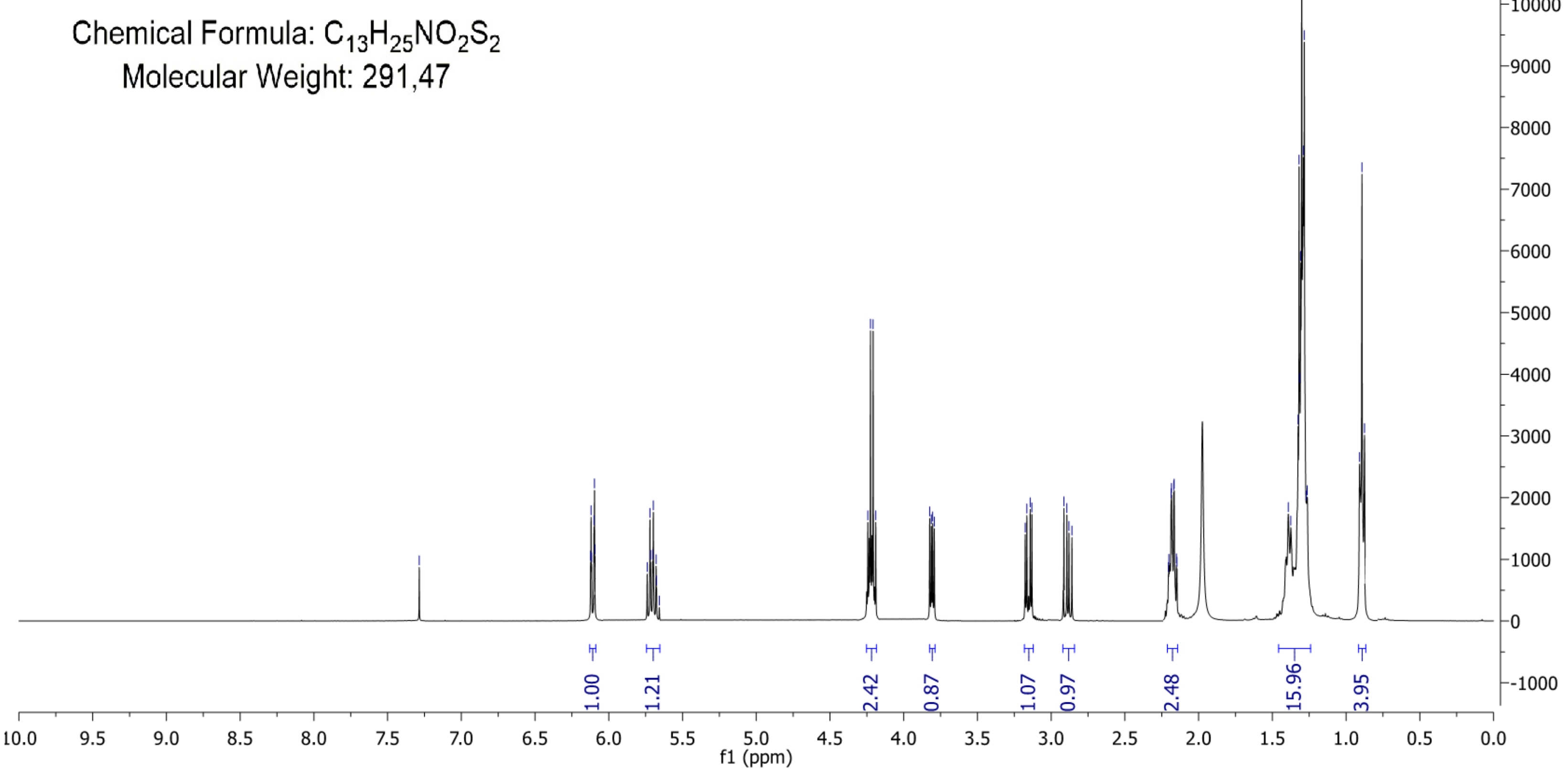

Z-6k

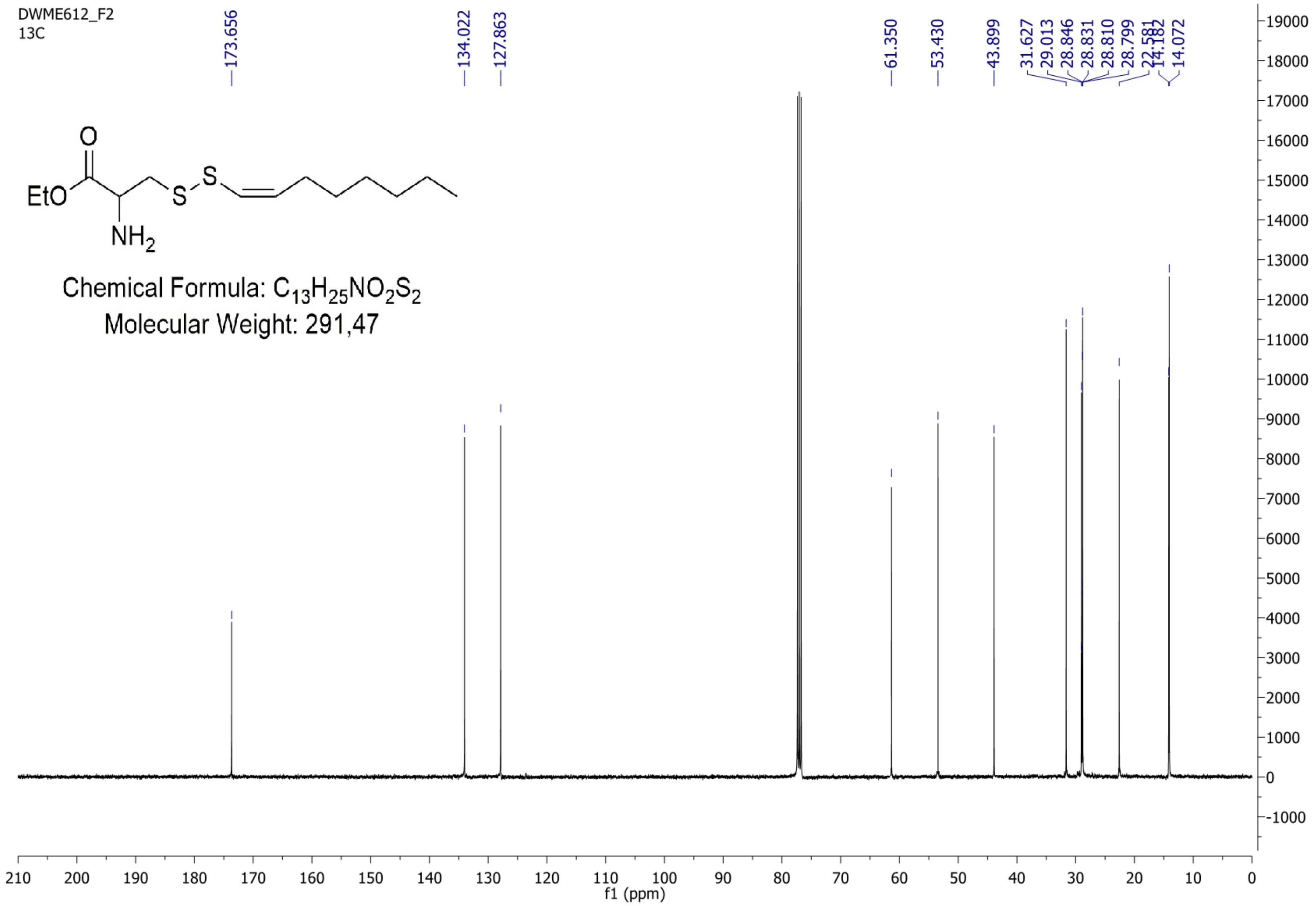

DWME612_F2
13C
173.656
134.022
127.863
61.350
53.430
43.899
31.627
29.013
28.846
28.831
28.810
28.799
22.581
14.182
14.072
EtO
O
S
S
NH2
Chemical Formula: C13H25NO2S2
Molecular Weight: 291,47
f1 (ppm)

IR (ATR) **Z-6l**

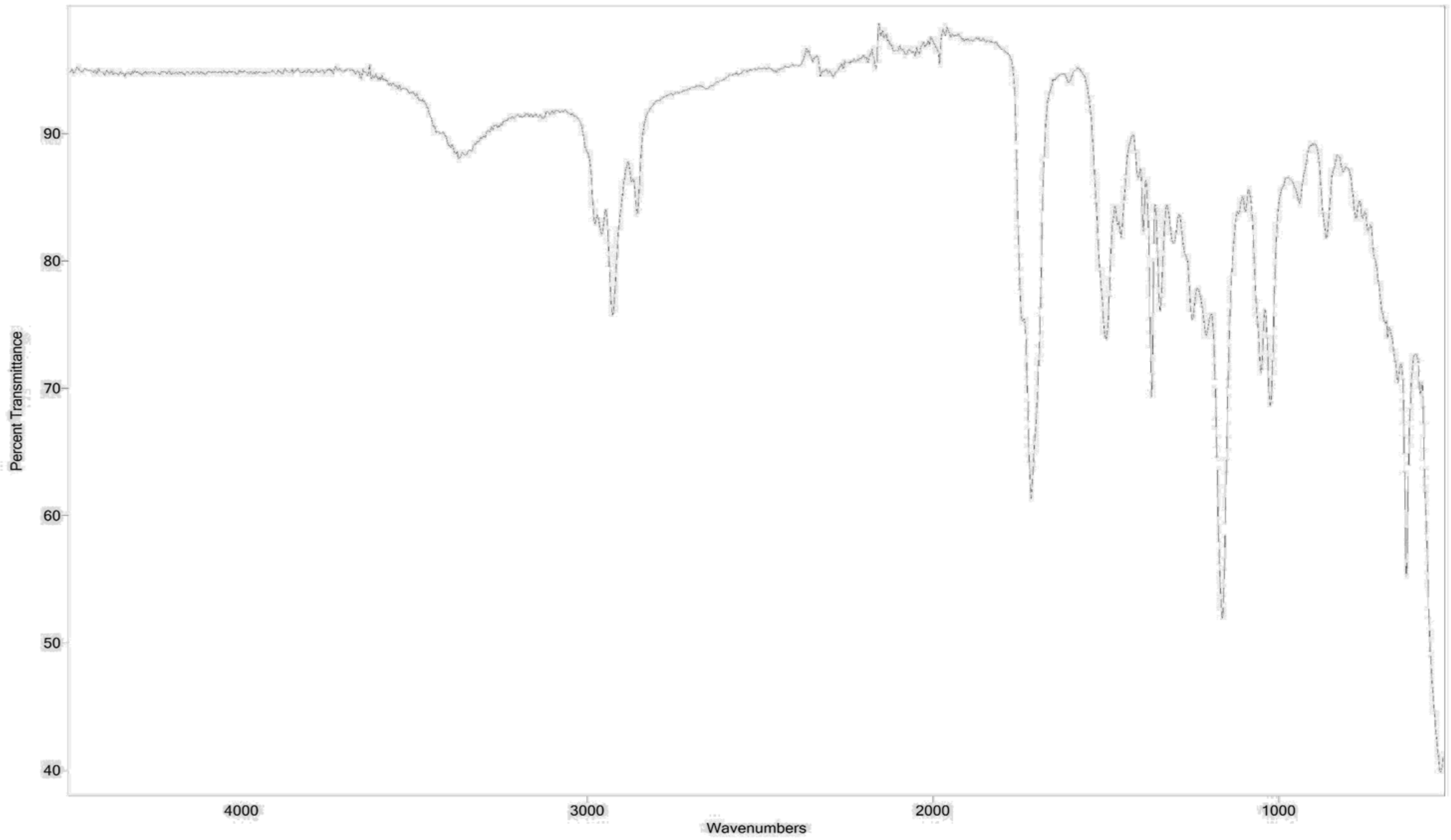

Percent Transmittance
90
80
70
60
50
40
4000
3000
2000
1000
Wavenumbers

Z-6l

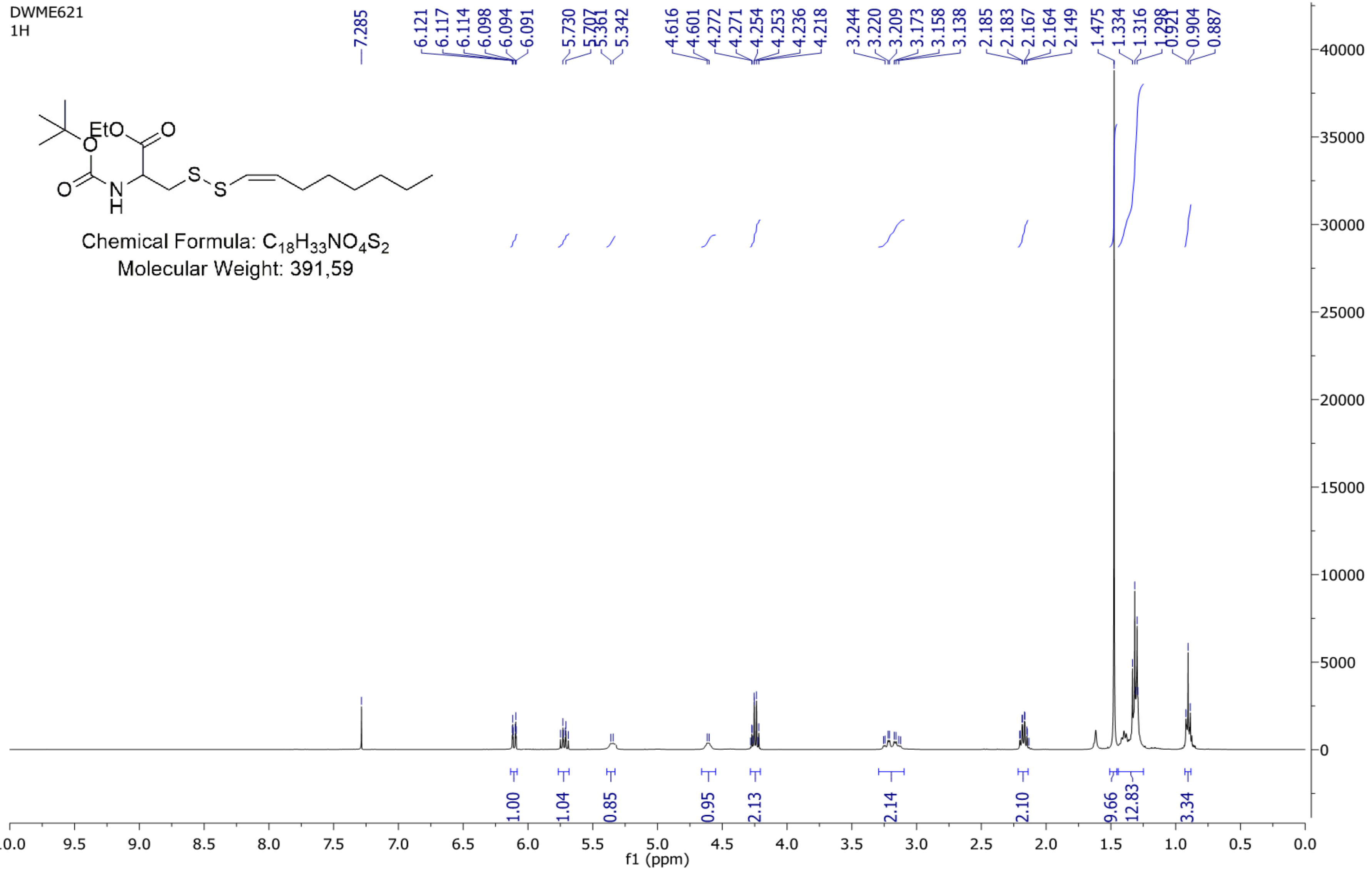
DWME621
1H
Chemical Formula: C18H33NO4S2
Molecular Weight: 391,59
f1 (ppm)

Z-6l

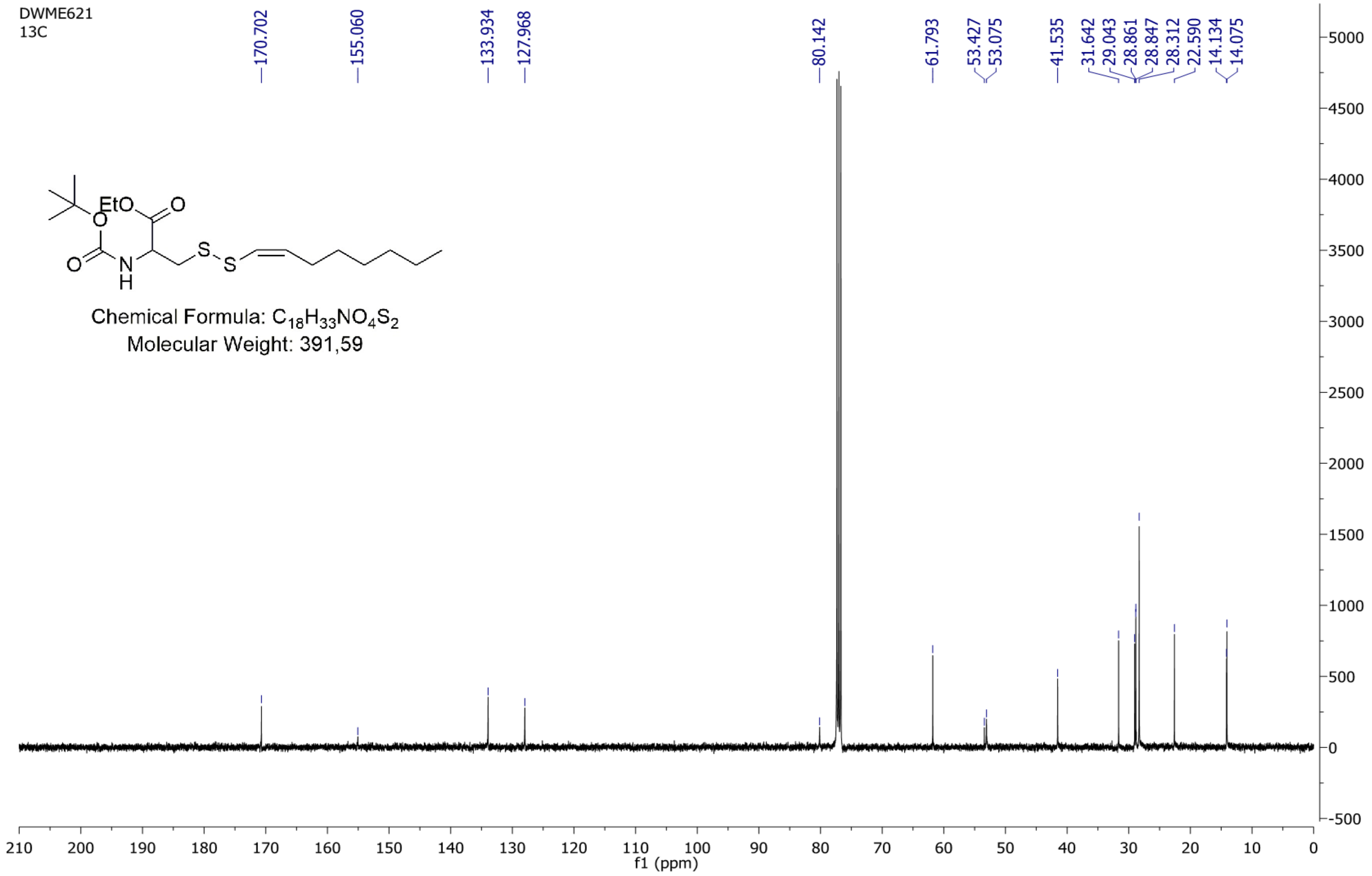
DWME621
13C
170.702
155.060
133.934
127.968
80.142
61.793
53.427
53.075
41.535
31.642
29.043
28.861
28.847
28.312
22.590
14.134
14.075
EtO
O
O
O
N
H
S
S
Chemical Formula: C18H33NO4S2
Molecular Weight: 391,59
f1 (ppm)

IR (ATR) **Z-6m**

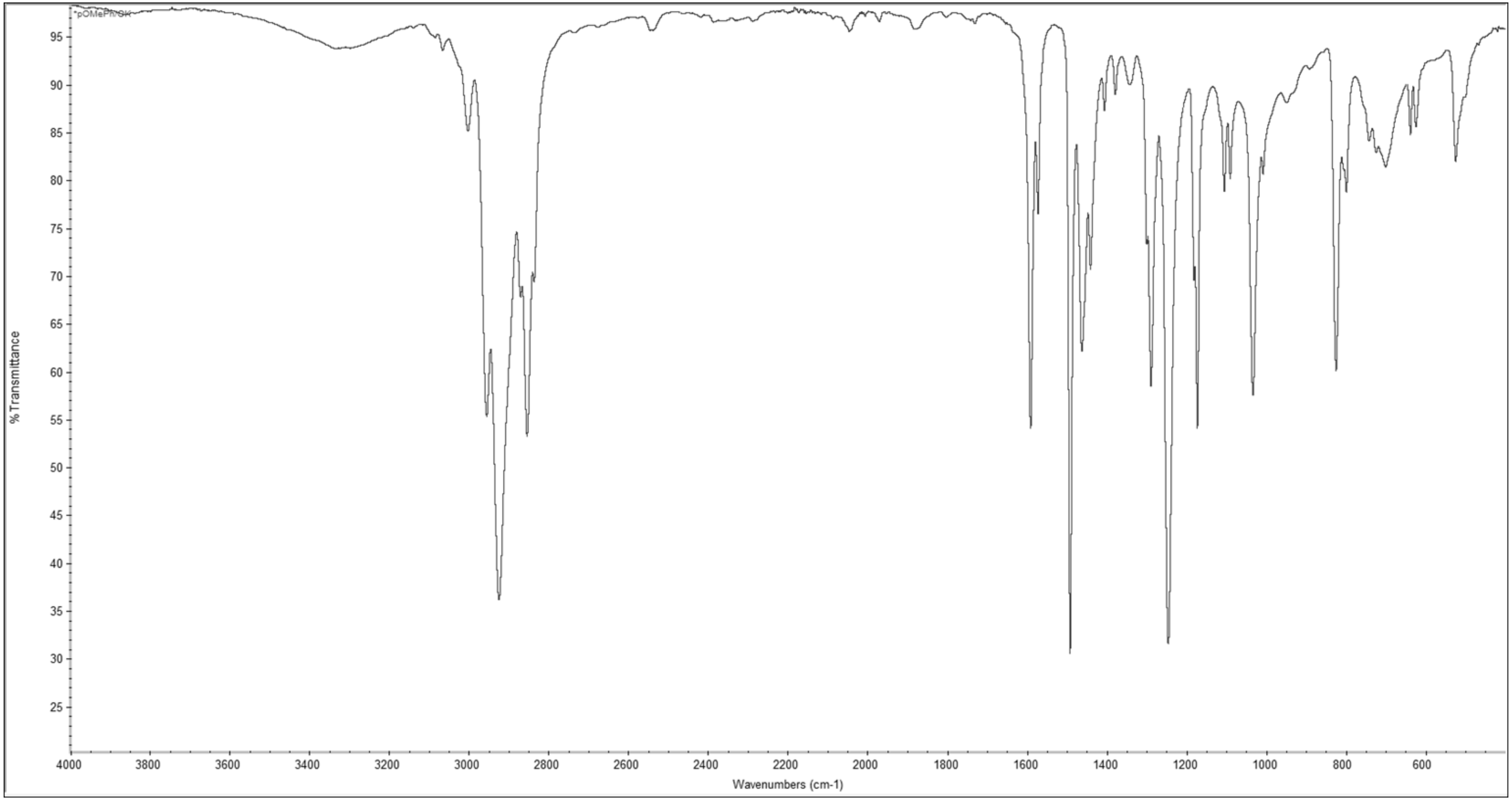

*pOMePh/GK
% Transmittance
95
90
85
80
75
70
65
60
55
50
45
40
35
30
25
4000
3800
3600
3400
3200
3000
2800
2600
2400
2200
2000
1800
1600
1400
1200
1000
800
600
Wavenumbers (cm-1)

Z-6m

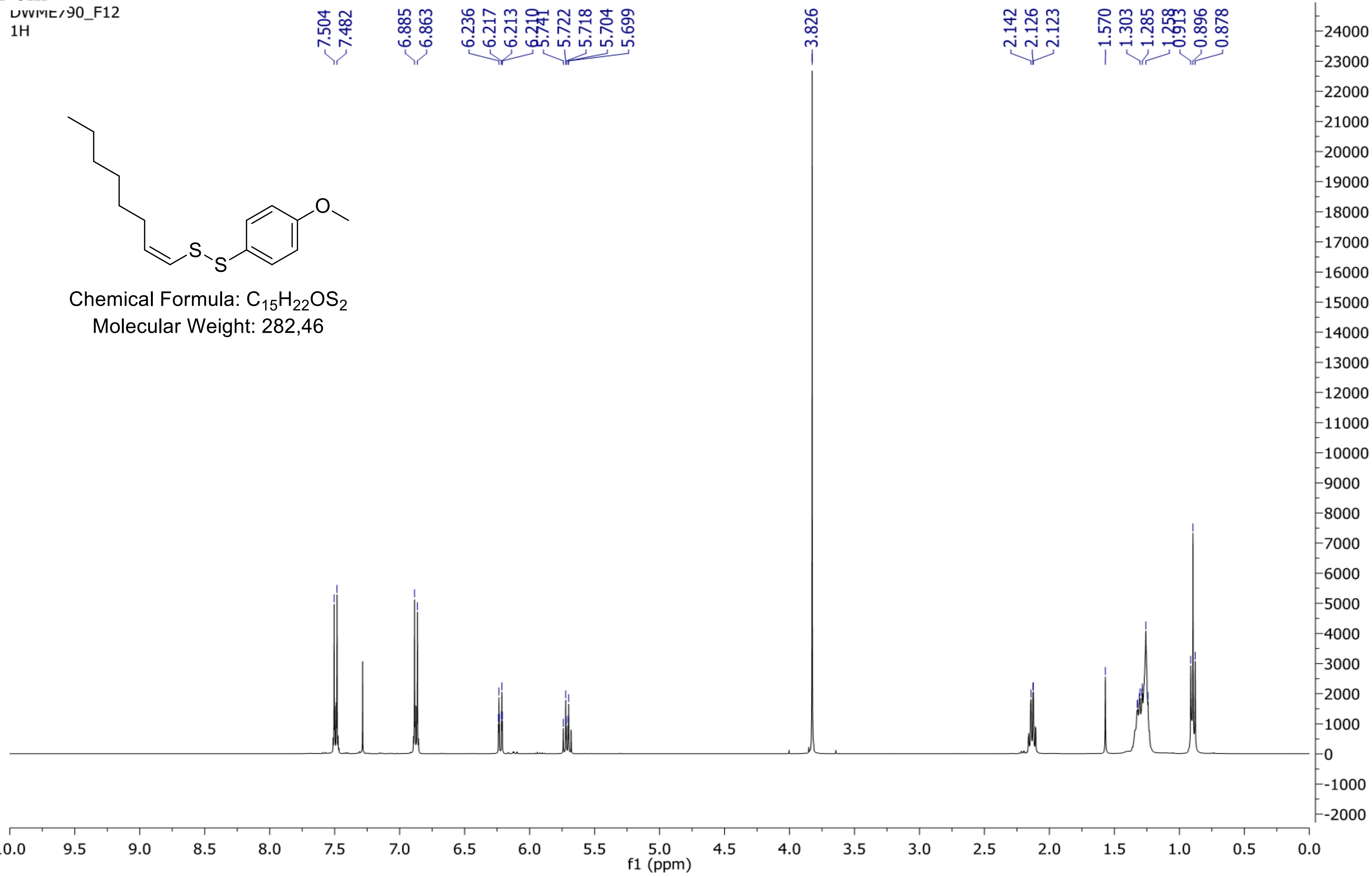

Chemical Formula: $C_{15}H_{22}OS_2$
Molecular Weight: 282,46

Z-6m

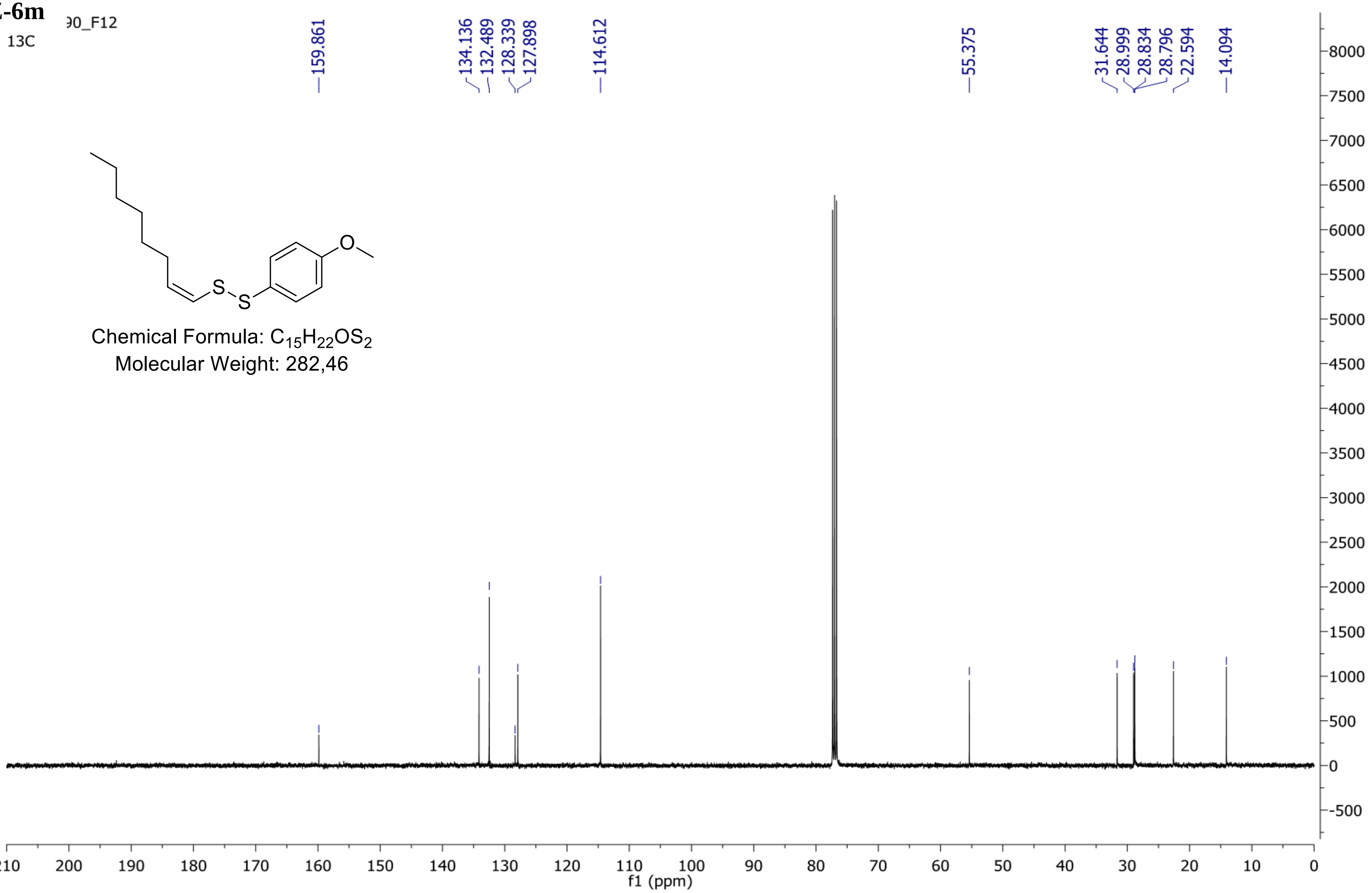

90_F12
13C
159.861
134.136
132.489
128.339
127.898
114.612
55.375
31.644
28.999
28.834
28.796
22.594
14.094
O
S
S
Chemical Formula: C15H22OS2
Molecular Weight: 282,46
f1 (ppm)
